# Sample tracking in microbiome community profiling assays using synthetic 16S rRNA gene spike-in controls

**DOI:** 10.1038/s41598-018-27314-3

**Published:** 2018-06-14

**Authors:** Dieter M. Tourlousse, Akiko Ohashi, Yuji Sekiguchi

**Affiliations:** Biomedical Research Institute, National Institute of Advanced Industrial Science and Technology, Tsukuba, Japan

## Abstract

Workflows for microbiome community profiling by high-throughput sequencing are prone to sample mix-ups and cross-contamination due to the complexity of the procedures and large number of samples typically analyzed in parallel. We employed synthetic 16S rRNA gene spike-in controls to establish a method for tracking of sample identity and detection of cross-contamination in microbiome community profiling assays based on 16S rRNA gene amplicon sequencing (16S-seq). Results demonstrated that combinatorial sample tracking mixes (STMs) can be reliably resolved by Illumina sequencing and faithfully represent their sample of origin. In a single-blinded experiment, addition of STMs at low levels was shown to be sufficient to unambiguously identify and resolve swapped samples. Using artificial admixtures of individually SMT-tagged samples, we further established the ability to detect and quantify cross-contamination down to a level of approximately 1%. The utility of our technique was underscored through detection of an unplanned case of cross-contamination that occurred during this study. By enabling detection of sample mix-ups and cross-contamination throughout 16S-seq workflows, the present technique thus assures provenance of sequence data on a per-sample basis. The method can be readily implemented in standard 16S-seq workflows and its routine application is expected to enhance the reliability of 16S-seq data.

## Introduction

High-throughput sequencing (HTS) is widely used to profile microbiota on an unprecedented scale, with research and diagnostic laboratories now routinely handling hundreds to thousands of samples. To fully exploit the vast throughput of today’s HTS platforms, workflows for microbiota profiling typically involve concurrent processing and analysis of tens to hundreds of samples by multiplexed sequencing^1,2^. Given the complex multistep procedures involved, this leads to a substantial risk of sample mix-ups and cross-contamination, especially in amplicon-based assays that employ two sequential PCRs for library construction^3,4^. Without dedicated methods, sample mix-ups and cross-contamination are challenging to identify and often remain undetected, as is evident from estimates indicating that up to 0.3–3% of HTS data may be compromised due to provenance errors^5,6^. While the prevalence of sample mix-ups and cross-contamination in microbiome studies is difficult to estimate, it is reasonable to presume that comparable levels of provenance errors occur, especially considering that multiple operators and laboratories are often involved in sample handling and processing in large-scale microbiome projects. When unidentified, sample mix-ups and cross-contamination may lead to invalid conclusions in research settings and compromise patient safety when generated data are used for actionable diagnostics.

Stringent laboratory procedures and workflow automation are effective measures to reduce the risk of sample swaps and cross-contamination but cannot guarantee complete elimination of errors. Additional quality control is thus required to detect potential sample swaps and cross-contamination. Because errors can arise at all steps of HTS workflows, including during sequencing and bioinformatics analysis^7^, methods that enable *post hoc* verification that all sequence data have been correctly assigned to their intended sample of origin are therefore recommended^8^. To promote widespread uptake and routine usage in the microbiome field, such techniques should be easy to implement in standard HTS workflows, without drastically increasing the cost or complexity of the analysis.

**Figure 1:**
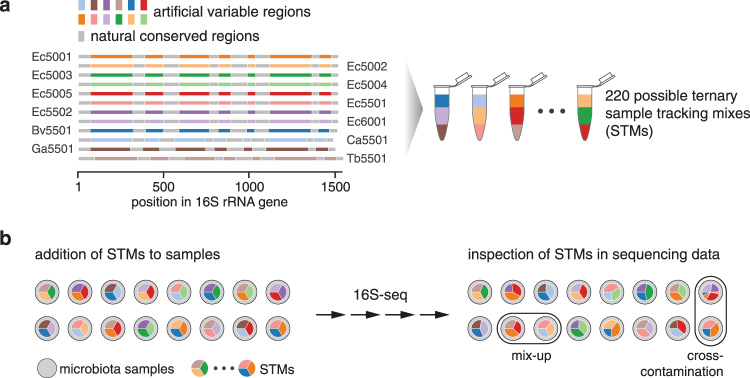
(**a**) Schematic of the synthetic 16S rRNA gene spike-in controls and formulation of combinatorial STMs for sample tracking. (**b**) Illustration of the utility of STMs for sample tracking based on spiking of individual samples with unique STMs at the beginning of the 16S-seq workflow.

In this study, we have developed a spike-in control method for sample tracking and detection of cross-contamination in microbiome community analysis by 16S rRNA gene amplicon sequencing (16S-seq). Such techniques have previously been described for microarrays and HTS assays^9,10^ but these have, to the best of our knowledge, not yet been adopted for 16S-seq. In short, the present method employs uniquely identifiable synthetic 16S rRNA gene spike-in controls that are added to samples at the beginning of the analytical workflow, with each sample receiving a distinct spike-in or mixtures of spike-ins (Fig. 1b). Once added to the samples, spike-in controls are carried through the entire analytical process, from sample processing to bioinformatics analysis, along with their accompanying sample. This ensures that sample mix-ups and cross-contamination that may introduced at any step can be identified in the resultant sequence data based on the presence of unplanned spike-in controls in a given sample (Fig. 1b). Using synthetic 16S rRNA gene spike-in controls previously developed by our group^11^, we designed and validated a set of ternary sample tracking mixes (STMs) and demonstrated their utility for resolution of sample swaps and detection of cross-contamination.

## Results

To establish a method for sample tracking in large-scale 16S-seq experiments, we used synthetic spike-in controls that are added to each sample prior to analysis, with a unique spike-in control mixture being incorporated into each sample. As detailed previously^11^, each spike-in control represents a near-full-length 16S rRNA gene sequence with *in silico* designed variable regions with negligible identity to nucleic acid sequences in public databases. For tracking of tens to hundreds of samples using a limited number of unique spike-in controls, we combined multiple spike-ins in equimolar mixtures to generate so-called sample tracking mixes (STMs). Here, STMs contained three different spike-ins each (Fig. 1a); starting from a set of 12 unique spike-ins, this theoretically accommodates 220 possible STMs. Based on this set, we carefully selected 96 STMs to be arranged in a standard multi-well plate, such that no individual spike-in controls were shared between row- and column-wise adjacent wells (Supplementary Table S2 and Fig. S1).

### Validation of ternary STMs

As a basic validation, amplicon libraries were prepared for all 96 STMs with primer set 338F-519R and sequenced on the Illumina NextSeq 500 platform, generating 2 × 150 bp paired-end reads. Following library demultiplexing and processing, reads were annotated by direct comparison against the spike-in sequences using USEARCH’s uparse_ref command.

Based on the proportion of total reads assigned to unused Nextera XT index combinations following demultiplexing, the rate of sample misassignment was estimated to be approximately 0.0014% when no mismatches were allowed in the index reads (Supplementary Fig. S2). As discussed in the Methods section, this represented a nearly 10-fold reduction as compared to permitting single index mismatches, which is the default settings used by Illumina’s bcl2fastq conversion software. Remaining sample misattribution may be due to contamination of indexed primers used for library barcoding and/or cross-talk introduced during sequencing, as a result of erroneous base calls in the index reads and/or mixed cluster formation on the flow cells^7^. We note that in our indexing scheme, unused indexes represent combinations for which either the i5 or i7 indexes were used for other samples in the same sequencing run, as recommended previously^12^. For a given sequencing run, the estimated level of cross-talk due to sample misattribution as determined based on unused indexes sets a lower bound on the degree of cross-contamination that can be detected.

**Figure 2:**
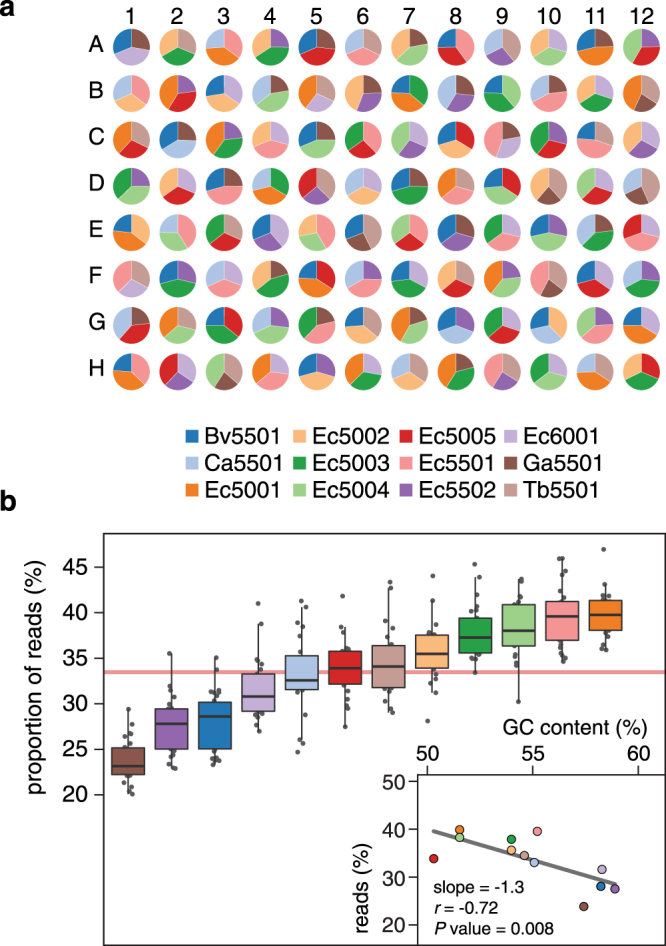
Experimental validation of 96 ternary STMs. (**a**) Pie charts showing the distribution of reads assigned to each of the spike-in controls across STMs. Note that reads assigned to unexpected spike-in controls for a given STM are also included but not visible due to their low proportional abundance. STMs are ordered as shown in Supplementary Fig. S1. (**b**) Boxplots of the distribution of normalized read counts for each of the spike-in controls across STMs. The horizontal red line represents the expected equal proportion of 33.3%. The inset shows the relationship between amplicon G+C content and normalized read count (mean across STMs for each of the spike-in controls).

A total of 29,527,812 processed reads were obtained across STM libraries; of these, 29,447,030 (99.7%) could be assigned to the spike-in sequences at 97% sequence identity. Across STMs, expected sequences were identified with proportional abundances ranging from 23.9 ± 2.3% (mean ± standard deviation) for Ga5501 to 39.9 ± 2.6% for Ec5001 (Fig. 2a,b), as compared to the expected value of 33.3%. Differences between expected and observed STM compositions were presumed to be due to PCR bias, a common source of inaccuracy in 16S-seq measurements^13,14^. Analysis of the abundances of each spike-in control across STMs further revealed a negative relationship between detection rate and amplicon G + C content (Pearson’s *r* = −0.72, *P* value = 0.008; Fig. 2b). This indicated that G + C content contributed to the perceived underestimation of specific sequences, consistent with its known effect on PCR efficiency^15^.

Compositional purity of each STM was expressed as the proportion of reads assigned to expected spike-in controls. As shown in Supplementary Table S4, purity across STMs was high, with a median of 99.997% and range of 100% (for stm12, 19, 66, 72 and 80) to 99.94% (for stm75). We evaluated whether contamination of stock solutions of individual spike-in controls may be responsible for the increased proportion of unexpected spike-in reads in some of the STM libraries. Based on co-occurrence analysis, no cases could be identified in which higher levels of contaminants were associated with the presence of a specific spike-in control (Supplementary Fig. S4). This suggested that contamination of stock solutions for individual spike-in controls was largely negligible. Moreover, levels of unexpected spike-ins for STMs processed individually in tubes were comparable to those of STMs processed simultaneously in 96-well plates (Supplementary Fig. S5). This verified that cross-contamination among STMs introduced during library preparation was minimal. Based on these observations, we concluded that between-STM contamination was likely introduced during manual mixing of the STMs in 96-well plates. Contamination levels were however sufficiently low as not to impact the accuracy of sample identification. This is because STMs will in their typical usage scenario be sequenced at much lower depth and low-level contaminants will hence remain undetected.

### Identification of swapped and cross-contaminated samples

We next performed a set of experiments to validate the performance of STMs for: (i) identification and resolution of sample mix-ups/swaps, and (ii) detection and quantification of sample cross-contamination. In these experiments, STMs were added to soil DNA at a final concentration of 5 × 10^3^ total spike-in control copies per ng of DNA. This was expected to yield about 2.5% of spike-in reads in the sequencing data, based on the assumption that 1 ng of environmental DNA contains approximately 2 × 10^5^ PCR-amplifiable 16S rRNA gene copies^11^. In terms of analysis, reads from the spike-in controls were identified following *de novo* OTU clustering using the UPARSE pipeline.

To establish samples for the sample swap experiment, unique STMs were individually added to 16 aliquots of DNA extracted from a single soil sample. Tube labels were then switched to mimic swapping or mislabeling of samples; swaps were blinded to the researcher responsible for data analysis and interpretation. OTU tables generated by *de novo* clustering contained a total of 5,129,041 sequences (median of 214,340 reads per sample) and spike-in controls accounted for 0.09 ± 0.05% of reads across samples (Supplementary Table S5). This was lower than the expected value but did not compromise accuracy of STM/sample assignment, as demonstrated below.

**Figure 3:**
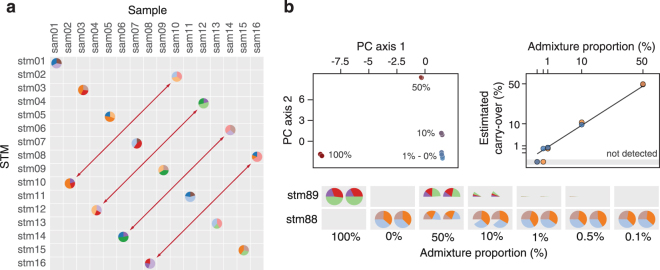
Demonstration of the utility of STMs for detection of sample swaps and cross-contamination. (**a**) Map illustrating the results of the sample swapping experiment. Samples (columns) and STMs (rows) are ordered such that pie charts located on the off-diagonal indicate swapped samples. Double-headed arrows highlight identified sample mix-ups, which were in all cases in agreement with the single-blinded experimental design (Supplementary Table S3). (**b**) Graphics summarizing the results of the cross-contamination experiment. The upper left panel displays the ordination of the samples, showing the expected trend based on the composition of the admixtures. The lower plot shows pie charts of the distribution of reads assigned to the STMs for both samples. The upper right panel shows the relationship between estimated carry-over and theoretical admixture proportions. The solid line shows the 1:1 diagonal. Data from technical replicates are shown.

Results of the sample swap experiment are shown in Fig. 3a. To visualize (dis)concordance between expected and observed STMs, we plotted a two-dimensional map of pie charts such that samples for which the observed STM agreed with the expected STM are located on the 1:1 diagonal. As shown, for 8 (out of 16) samples observed and expected STMs were in agreement, indicating that this set of samples was not swapped. For the other half of samples, expected and observed STMs were in disagreement; these samples were thus scored as switched/mislabeled. Identified swaps and their STM-based reassignments in all cases agreed with the (single-blinded) experimental design (Supplementary Table S6). Taken together, these data validated the use of STMs for sample identification and also underscored that only a minor fraction of reads needs to be sacrificed in order to allow unambiguous STM/sample identification. Finally, we note that only a single STM was identified in all sample swap libraries, indicating that cross-contamination for this experiment, if present, was below the limit of detection.

To establish samples for the cross-contamination experiment, two different STMs were added to DNA extracted from two contrasting microbiota, namely from sewage (DNA spiked with stm88) and soil (DNA spiked with stm89). Artificially contaminated samples were then prepared by mixing both STM-tagged samples in varying ratios, from 50% to 0.1% of soil DNA diluted in sewage DNA. Following analysis, OTU tables contained a total of 7,025,477 sequences (median of 499,727 reads per sample) and spike-in reads accounted for 0.6 ± 0.8% of reads in a given library (Supplementary Table S7).

Results of the sample cross-contamination experiment are summarized in Fig. 3b. Firstly, OTU-based ordination of the microbiota profiles was consistent with the composition of sample admixtures (Fig. 3b and Supplementary Fig. S6). Secondly, stm89 (derived from the soil-DNA sample) was identified consistently in duplicate libraries of sludge-DNA samples containing at least 1% of soil DNA (Fig. 3b). Contamination of 0.1% was below the limit of detection as no reads for stm89 were detected in the corresponding samples. Thirdly, the ratio of reads assigned to the majority and minority STMs, stm88 and stm89 respectively, were in good agreement with theoretical sample mixing ratios (Fig. 3b). This suggested that levels of cross-contamination or carry-over can be quantified based on the proportion of reads assigned to the majority/minority STMs. These estimates should however be interpreted with caution since they may be skewed due to PCR bias resulting in uneven amplification among spike-in controls, as discussed above. As a whole, these data validated the utility of STMs for detection and quantification of between-sample cross-contamination.

It should be noted that when using mixtures of multiple spike-in controls, sources of cross-contamination might not always be uniquely resolvable. For the set of 96 STMs described in this study, we calculated that for two-sample cross-contamination, 59 ± 5% of possible pairwise cross-contamination events should be uniquely resolvable to a single source while 29 ± 6% will be attributable to two potential samples (Supplementary Fig. S7). For contamination involving three samples, the proportion that may be uniquely resolved is reduced to 0.7 ± 0.1% across STMs.

### Case demonstration

To further validate our method, we next evaluated performance of the STMs when added directly to samples at the point of DNA extraction. To this end, we prepared a set of samples by adding unique STMs to 16 aliquots of a single soil sample and performed automated DNA extraction using a robotic liquid handling system. Four unspiked soil samples and non-template controls for DNA extraction were also included (see Supplementary Fig. S8 for layout of the 96-well plate). We additionally prepared 16 samples consisting of soil DNA spiked with unique STMs and 4 unspiked soil DNA samples. These samples were added to the 96-well plate with automatically extracted DNA samples; all samples were then simultaneously subjected to PCR. Four non-template controls for PCR were also included.

Across samples, a total of 5,120,013 reads were recovered in the OTU table (median of 76,483 reads per sample) and spike-in controls accounted for 2.3 ± 1.0% and 1.8 ± 0.5% of reads for the spiked soil and soil-DNA samples, respectively (Supplementary Table S9). For the non-template controls, libraries were dominated by OTUs affiliated with *Methylobacterium* spp. (DNA extraction controls) and *Comamonadaceae* and *Sphingomonadaceae* (PCR controls) (Supplementary Table S11), consistent with common kit and reagent contaminants reported in other studies^16,17^. No STMs were identified in any of the NTC libraries. Based on the majority STMs identified for each of the STM-tagged libraries (*n* = 32), no sample swaps were identified (Supplementary Fig. S9 and Table S10). Furthermore, for the unspiked samples, individual spike-in controls were detected by only single reads individually. These accounted for at most 0.0025% of reads in a given library, comparable to the background level of sample cross-talk due to index misassignments.

**Figure 4:**
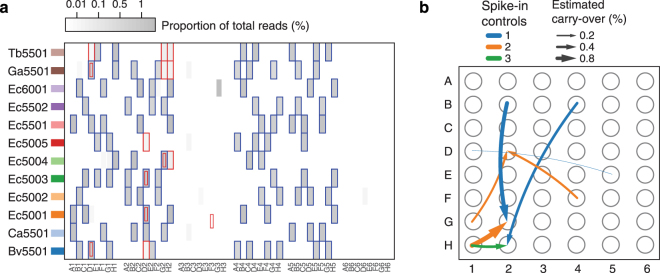
Evaluation of the STMs added at the point of DNA and case demonstration of unanticipated cross-contamination. (**a**) Heatmap of normalized read counts for each of the spike-in controls in the different samples. Blue and red boxes indicate spike-in controls assigned to the majority and minority STMs for each sample, respectively. Note that spike-in controls may be marked with both blue and red boxes in cases where spike-in controls are shared between the majority and minority STMs. (**b**) Visualization of the inferred pattern of cross-contamination based on minority STMs. The width of the arrows is scaled according to the estimated percentage of between-sample carry-over. Arrows are colored based on the number of non-shared spike-in controls between the source and contaminated samples/wells.

All libraries prepared from samples spiked at the DNA level were scored as ‘uncontaminated’ since no minority STMs were present in the sequencing data (Fig. 4a and Supplementary Table S9). Unexpectedly, we found that a small number of libraries prepared from samples spiked prior to DNA extraction contained minority STMs indicative of cross-contamination, namely for samples/wells D1, D2, G2 and H2 (Supplementary Table S9). The highest levels of minority STMs were present in samples G2 and H2 (Fig. 4a), with contaminating spike-in controls accounting for 0.9% and 0.5% of total spike-in reads for samples G2 and H2, respectively. Based on the identity of the minority STMs, potential sources of cross-contamination could be identified. Although source tracking was not unambiguous, sample/well H1 was identified as a common source of contamination for both samples G2 and H2. Carry-over from sample H1 was estimated to be approximately 0.5% to 1.5% (Fig. 4b and Supplementary Table S10), supported by two (for sample G2) or three (for sample H2) non-shared spike-in controls between the majority and minority STMs. Although it is not possible to fully resolve how the cross-contamination had been introduced, it seems reasonable to attribute the comparatively high level of contamination to a splash that occurred during the DNA extraction step. For samples D1 and D2, minority STMs were only supported by one or two reads (Supplementary Table S9). While potential sources of contamination could be identified (Fig. 4b), confidence in these calls was low due to the small numbers of reads recovered for the minority STMs.

## Discussion

Sample mix-ups and cross-contamination represent a potentially substantial source of errors in HTS assays. Quality control to verify data provenance is therefore critical to ensure validity of the results. Here we demonstrated that addition of synthetic 16S rRNA gene spike-in controls to microbiota samples provides a straightforward and easy-to-adopt technique for tracking of samples throughout 16S-seq workflows. Added spike-in controls are subjected to all experimental and computational steps along with the samples and allow sample mix-ups and cross-contamination introduced at any step to be identified in the resultant sequence data.

Using Illumina NextSeq 500 sequencing, we showed that combinatorial mixtures of spike-in controls can be reliably resolved and remain closely associated with their original sample throughout 16S-seq workflows, from DNA extraction to bioinformatics analysis. Combinatorial mixtures provide the chief benefit that a large number of unique STMs can be generated starting from a relatively small number of individual spike-in controls, thereby reducing cost and logistics challenges. Based on the set of 12 spike-in controls used in this study, up to 220 unique ternary STMs are possible while as many 924 can be generated when combining 6 spike-in controls per STM. Because the number of possible STMS grows very rapidly with the number of individual spike-in controls, only 16 spike-in controls would be needed to generate a pool of >10,000 unique STMs, each containing 7 spike-in different controls. Depending on the user’s requirements for STM layouts (for example, neighboring STMs should not share spike-in controls), this should be sufficient to generate hundreds to thousands of multi-well plates.

An important benefit of using synthetic spike-in controls is that cross-contamination can be detected with high sensitivity. We showed the ability to detect and quantify two-sample cross-contamination with a limit of detection of ~1%. Sensitivity could be easily improved by increasing STM spike-in levels. For example, increasing spike-in amounts ten-fold should theoretically enable ten-fold lower levels of cross-contamination to be detected at a given sequencing depth.

The amount of STMs added to samples can be chosen by the user based on the desired number of reads for the spike-in controls and the proportion of sequence data that can be sacrificed. In our experiments, only a few tens to hundreds of spike-in reads proved sufficient to robustly identify samples and resolve swapped samples. As such, a spike-in level on the order of 1% (by total 16S rRNA gene copies) may be recommended to be sufficient in many scenarios. At a sequencing depth of 30,000 reads per sample, which is commonly achieved in microbiome studies, this is expected to yield around 300 reads per STM or approximately 100 reads per spike-in controls when using ternary STMs and assuming minimal detection bias.

In our study, we observed that standard Illumina index reagents can provide low sample misassignment rates using the NextSeq 500 sequencing platform. Still, high-purity reagents (such as TruGrade DNA Oligos from Integrated DNA Technologies) could be beneficial to address the risk of manufacture cross-contamination of index stock solutions^9,18^. In addition, demultiplexing algorithms that take into account index read quality^12^ and/or probabilistically model differences between observed and true index sequences based on index read Phred scores^18^ can be explored in cases where filtering based merely on index mismatches results in the loss of too many correctly assigned reads or is ineffective.

The unplanned case of cross-contamination that we encountered in this work underscores the necessity to pay sufficient attention to issues related to cross-contamination. At a minimum, it is recommended to assess the performance of new experimental workflows or when changes are made to existing workflows, preferably using a comprehensive set of STMs that are added to a large set of samples. Depending on the need of the user, routine implementation of the spike-in controls for sample assurance may take different forms. Rather than barcoding of all samples, the user may for example prefer to distribute a limited number of dedicated spike-in control samples across a multi-well plate and track these across all other unspiked samples processed and sequenced in parallel. This allows estimation of background levels of cross-contamination^19^, which should be useful for establishing read count thresholds to confidently assign identified taxa as present in a given sample.

## Conclusion

We described a synthetic spike-in control method for sample tracking in 16S-seq assays. This technique allows final sequence data to be vetted for sample swaps and/or cross-contamination and hence provides an added level of confidence in data provenance for each individual sample. As described, the method can be readily adopted for applications in microbiome research settings. With further validation, the technique could also be used for diagnostics applications where robust detection and resolution of sample/data provenance issues are critical.

## Methods

### Synthetic 16S rRNA gene spike-in controls

The design and preparation of the synthetic 16S rRNA gene spike-in controls were detailed previously^11^ (see Supplementary Table S1). Spike-in controls were prepared as linearized plasmid DNA (10 ng/μl in Tris-EDTA buffer, pH 8.0) and STMs were formulated by mixing stock solutions of individual DNA plasmids in 96-well plates. The latter were sealed with individual strip caps to provide access to individual columns in order to reduce the risk of cross-contamination.

### Environmental samples and DNA extraction

Soil and sewage samples were collected from a small forest at the premises of the National Institute of Advanced Industrial Science and Technology (AIST) and a municipal wastewater treatment plant, respectively, both in Ibaraki (Japan). Manual DNA extractions were performed using the FastDNA SPIN Kit for Soil and FastPrep Instrument (MP Biomedicals), following the manufacturer’s instructions. If applicable, the Eppendorf epMotion M5073 liquid handling workstation was used for automated DNA extraction using the PowerMag Microbiome RNA/DNA Isolation Kit (MO BIO Laboratories), according to the provided protocols. Integrity of the extracted DNA was verified by agarose gel electrophoresis and concentrations determined with the Quant-iT dsDNA High-Sensitivity Assay Kit using a Qubit 3.0 fluorometer (Thermo Fisher Scientific). DNA extracts were stored in nuclease-free H_2_O at −20 °C until use.

### Amplicon library preparation and Illumina sequencing

Sequencing libraries for the V3 hypervariable region of the 16S rRNA gene were prepared by two-step tailed PCR following Illumina’s “16S Metagenomic Sequencing Library Preparation” protocol^20^. Unless stated otherwise, PCR reactions were set up in 96-well plates using the EpMotion M5073 liquid handling system. The first round of PCRs (20 μl) contained 1 × PCR Gold buffer, 1.5 mM of MgCl_2_, 200 μM of each deoxynucleotide triphosphate, 0.5 units of AmpliTaq Gold LD DNA polymerase (all from Applied Biosystems), 500 nM each of forward (356 F: 5′-ACWCCTACGGGWGGCWGC-3′) and reverse (519 R: 5′-GWATTACCGCGGCKGCTG-3′) primer (synthesized by Tsukuba Oligo Services, Tsukuba, Japan) and 2 μl of template DNA. Template DNA consisted of 2 × 10^5^ total copies of spike-in controls or approximately 2 ng of (spiked) environmental DNA. Primer sequences contained appropriate 5′-end adapters for indexing in the second round of PCR. Thermal cycling conditions were as follows: 95 °C for 9 min, followed by 25 cycles of 95 °C for 45 s, 50 °C for 45 s and 72 °C for 1 min, and 72 °C for 5 min. PCR products were purified using the Agencourt AMPure XP system (1.5 × volume of AMPure beads) and eluted in 30 μl of nuclease-free H_2_O. The second round of PCRs (20 μl) contained 1 × KAPA HiFi HotStart ReadyMix (Roche), 2 μl each of i5 and i7 Nextera XT indexing oligos (Illumina) and 2 μl of purified first-round PCR product. Thermal cycling conditions were as follows: 95 °C for 3 min, followed by 8 cycles of 95 °C for 30 s, 55 °C for 30 s and 72 °C for 30 s, and 72 °C for 5 min. Following purification using 1.5 × AMPure beads, amplicons were pooled, and DNA concentration of the pooled library quantified using the High Sensitivity D1000 ScreenTape Assay system and 2200 TapeStation instrument (Agilent). The library was supplemented with 30% of PhiX DNA and sequenced on an Illumina NextSeq 500 instrument using the Mid-Output v2 Kit (300 cycles). The Real-Time Analysis software (RTA v2.4.6) was used for automated base calling and quality scoring. As implemented in the NextSeq 500 sequencing workflow, the instrument was automatically washed with dilute sodium hypochlorite following runs in order to mitigate library contamination due run-to-run sample carryover.

### Sequence read processing and analysis

Binary base call files were converted off-board to fastq format using Illumina’s bcl2fastq Conversion Software (version 2.16.0.10) with concurrent trimming of sequencing adapters and library demultiplexing based on the dual index reads. In preliminary analyses, it was found that allowing no mismatches in the index reads (bcl2fastq option “barcode-mismatches 0”) reduced the proportion of demultiplexed reads assigned to unused index combinations by one order of magnitude as compared the default settings of allowing one mismatch per index read (Supplementary Fig. S2). Using this setting, approximately 95% of raw reads assigned to expected index combinations were retained as compared to the default settings of allowing a single mismatch for index reads (Supplementary Fig. S3). As such, we set the “barcode-mismatches 0” option as the default in our bioinformatic pipeline in order to minimize apparent sample cross-contamination due to errors introduced during demultiplexing.

Demultiplexed read pairs were quality-filtered using Trimmomatic^21^ (version 0.32) with the following parameters: HEADCROP:6 SLIDINGWINDOW:15:20 MINLEN:75. Quality-filtered reads were then merged by USEARCH^22^ (version 8.1.1861), using the -fastq_mergepairs command with the following options: -fastq_minovlen 30 -fastq_maxdiffs 9999 -fastq_maxdiffpct 5. Primer sequences were subsequently trimmed using Cutadapt^23^ (version 1.8.3), allowing a single mismatch and requiring that the forward and reverse primers were anchored at the 5′ and 3′ end of the merged reads, respectively; untrimmed reads lacking identifiable forward and/or reverse primer sequences were discarded. A summary of the number of reads retained following each of the processing steps is provided in Supplementary Table S3.

For libraries constructed from samples containing only spike-in controls, reads were annotated using USEARCH’s uparse_ref command with default settings; non-chimeric hits with a global sequence identity of ≥97% were considered as matches and tallied to construct count tables for downstream analysis. For libraries constructed from spiked environmental microbiota, reads were clustered *de novo* into operational taxonomic units (OTUs) using the UPARSE pipeline^24^. Specifically, sequences were dereplicated (command -derep_fulllength) and sorted by abundance with concurrent removal of sequences less than four times in the entire (command -sortbysize with option -minsize 4). The latter threshold was chosen to account for the high sequencing depth achieved using the NextSeq 500 instrument. Cluster/OTU centroids were then determined using the -cluster_otus command with default parameters (otu_radius 3), including *de novo* chimera filtering. Additional reference-based chimera filtering of the OTU centroids was performed using -uchime_ref^25^ against the Broad Microbiome Utilities’ GOLD database (version microbiomeutil-r20110519, supplemented with the spike-in control sequences) with option -minh 1.0. Finally, OTU count tables were constructed by mapping all processed reads to the chimera-checked OTU centroids with the -usearch_global command (options -id 0.97 -leftjust -rightjust). Centroid sequences were compared to the Greengenes database^26^ (release 8_13) by BLAST and taxonomy assigned based on the top hit, using the QIIME^27^ (version 1.8.0) script parallel_assign_taxonomy_blast.py. Only a single OTU for each of the spike-in controls was identified for the spiked environmental microbiota samples (data not shown).

### Downstream data analysis

Downstream manipulation and analysis of OTU count tables was performed in the R statistical computing environment^28^; graphics were generated using the R package ggplot2^29^. Additional core packages used included magrittr^30^, tidyr^31^ and dplyr^32^ for data manipulation and vegan^33^ for community ecology analysis of the OTU tables. If applicable, OTU count tables were randomly subsampled to even depth using vegan’s rarefy function. Ordination analysis was performed based on rarefied and log_10_(x + 1)-transformed OTU count tables by principal components analysis (PCA) using vegan’s rda function.

Analysis of STM read counts for sample classification was performed as follows. For each of the libraries, we screened for the presence of all possible STMs used in a given experiment, with the requirement that all spike-ins for a given STM needed to be present (that is, supported by at least a single read) in order for the STM to be scored as present. At this point, samples with only a single STM were considered as uncontaminated. STM identifiers were then compared to the expected STMs to verify sample identity and resolve potential sample mix-ups. For libraries containing multiple STMs, the majority STM was defined based on the highest cumulative reads counts and the majority STM used to assign sample identity as above. Potential sources of cross-contamination were tracked based on the composition of the minority (that is, contaminating) STMs and quantified based on the proportion of spike-in reads assigned to the minority STMs, after exclusion of shared spike-in controls present in both the majority and minority STMs.

A custom R script to perform the above analyses is available in a dedicated GitHub repository (https://github.com/aistBMRG/SampleTrackeR). Raw data files and an R Markdown document are also provided to replicate the main results presented in our study.

### Availability of materials and data

The sequencing data (raw fastq files following processing by bcl2fastq) generated during the current study are available in the NCBI Sequence Read Archive under accession number SRP111031 (Bioproject PRJNA388158). Raw count data for the different experiments are provided in the Supplementary Information and are also provided as part of SampleTrackeR’s GitHub repository.

## Electronic supplementary material

Supplementary Information
