# Supplementary material for: Sample tracking in microbiome community profiling assays using synthetic 16S rRNA gene spike-in controls
Source: Sci Rep. 2018 Jun 14;8:9095. doi: 10.1038/s41598-018-27314-3 (PMC6002373; doi:10.1038/s41598-018-27314-3)
Supplement: Supplementary file 1 — Supplementary Information [file 41598_2018_27314_MOESM1_ESM.pdf]

Supplementary Information for

Sample tracking in microbiome community profiling assays using synthetic 16S rRNA gene spike-in controls

Dieter M. Tourlousse, Akiko Ohashi, Yuji Sekiguchi

Biomedical Research Institute, National Institute of Advanced Industrial Science and Technology, Tsukuba, Japan

Address correspondence to: Dieter Tourlousse, dieter.tourlousse@aist.go.jp

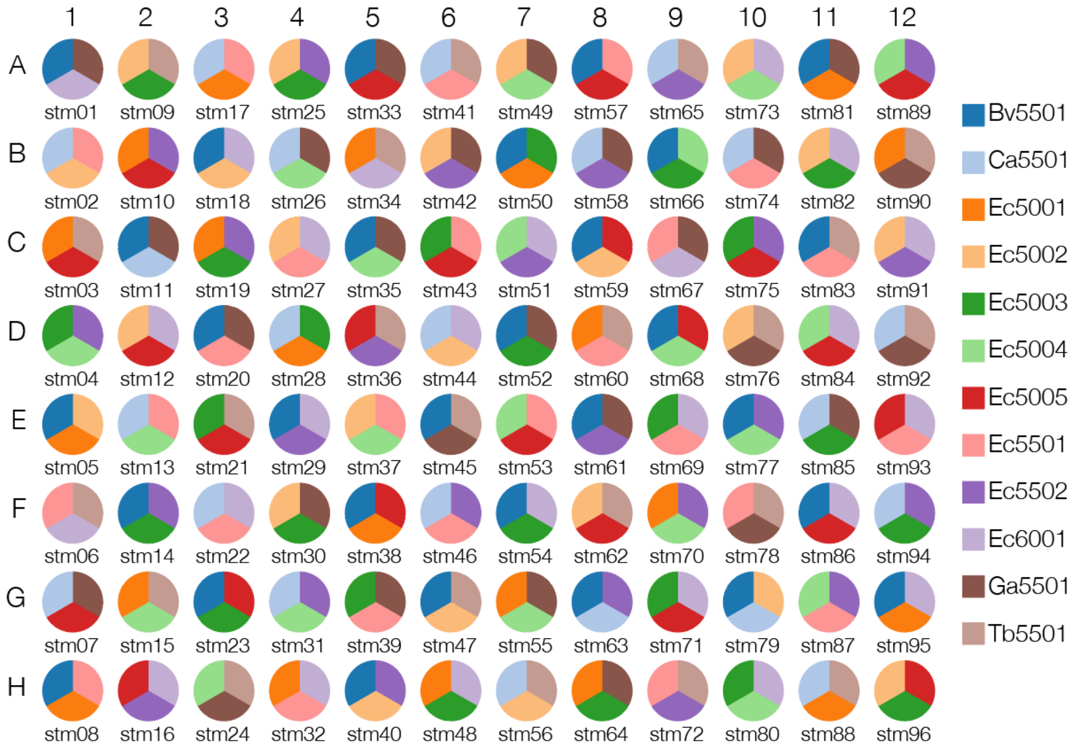

Figure S1. Layout of the 96 sample tracking mixes (STMs) in a standard multi-well plate. STMs were organized such that row- and column-wise neighboring wells shared no spike-in controls.

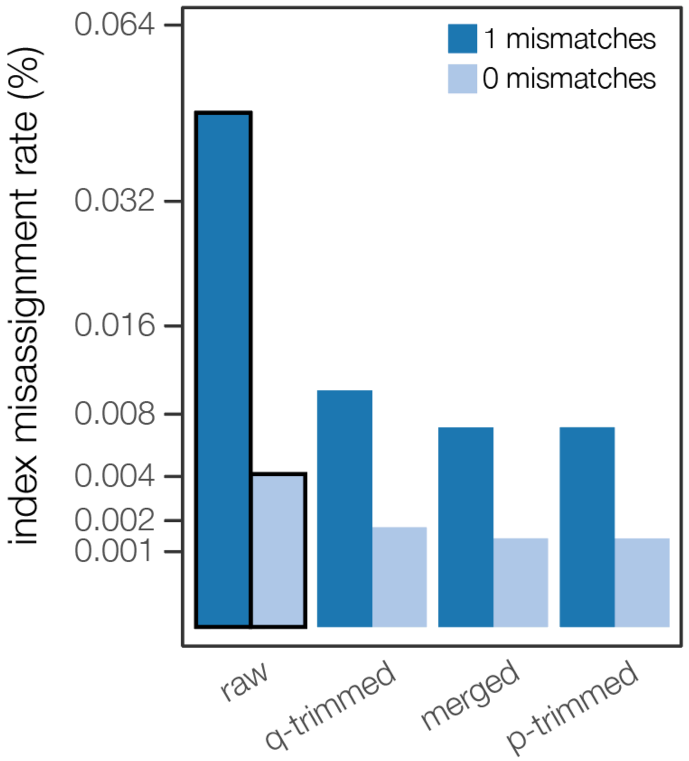

Figure S2. Index misassignment rates for reads demultiplexed by bcl2fastq with two different settings (0 index mismatches allowed: --barcode-mismatches 0 and 1 index mismatch allowed: --barcode-mismatches 1). Rates were estimated based on the proportion of demultiplexed reads assigned to unused index combinations at various steps of the bioinformatics workflow: raw, raw reads; q-trimmed, quality-filtered read pairs; merged, merged reads; p-trimmed, primer-trimmed reads. Raw read pairs are highlighted for clarity.

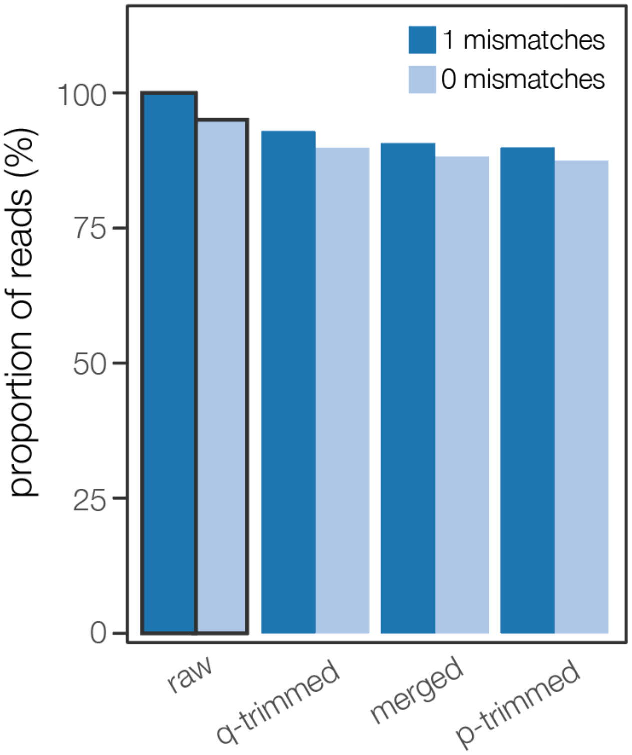

Figure S3. Relative number of reads retained at each of the read processing steps for reads demultiplexed by bcl2fastq with two different settings (0 index mismatches allowed: --barcode-mismatches 0 and 1 index mismatch allowed: --barcode-mismatches 1). Horizontal categories are as in Fig. S2. Raw read pairs are highlighted for clarity.

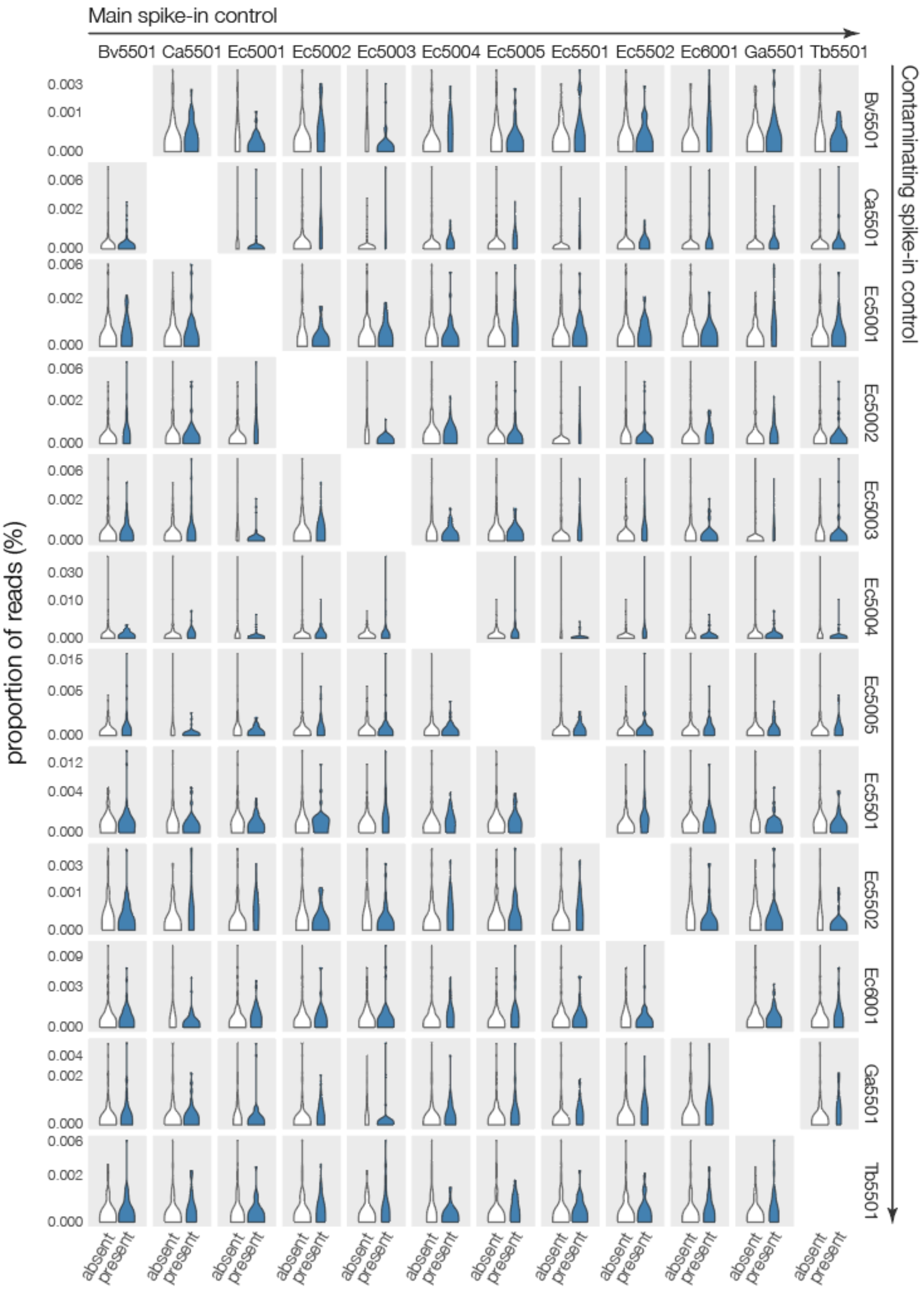

Figure S4. Screening for potential contamination of individual spike-in control DNA plasmid stock solutions based on co-occurrence analysis. For each spike-in controls (shown as columns), the proportional abundances of all other spike-in controls (shown as rows) in STMs for which the main spike-in control was either absent or present (categories on the x-axis) are plotted. Distributions were calculated using ggplot2's geom_violin function.

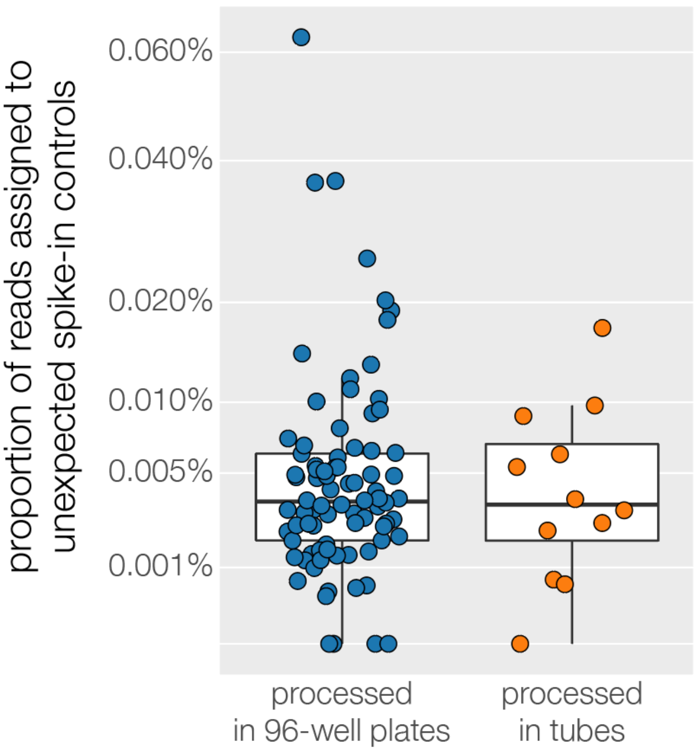

Figure S5. Proportion of spike-in control reads assigned to unexpected spike-in controls for STMs processed concurrently in 96-well plates as compared to STMs processed individually in tubes. Boxplots were generated using ggplot2's geom_boxplot function.

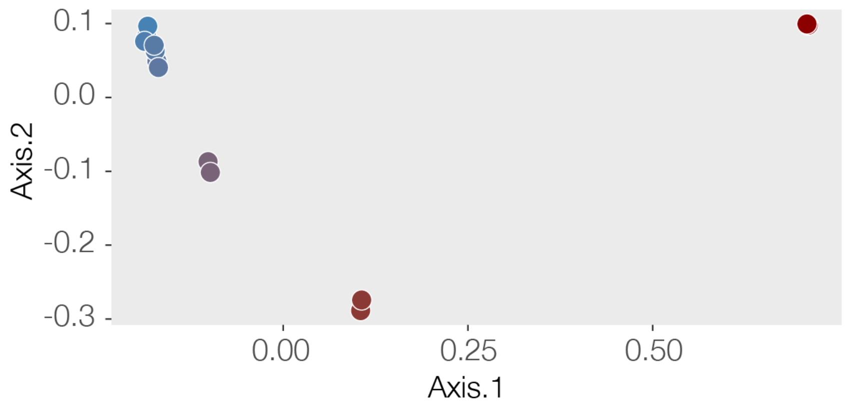

Figure S6. Ordination of samples from the cross-contamination experiment. Data represent principal coordinates analysis (PCoA) ordination based on the presence/absence Jaccard distance matrix. Symbols are colored according to the admixture ratios as in Fig. 3B in the main text.

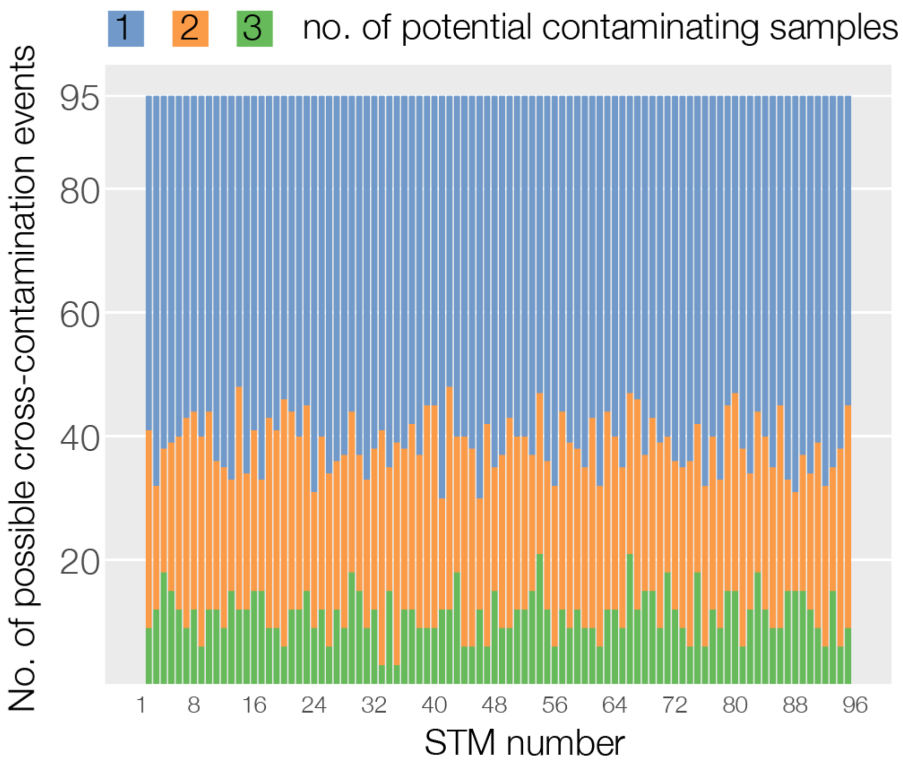

Figure S7. Bar chart showing the number of two-sample cross-contamination events that would theoretically be attributable to a unique source of contamination (no. of potential contaminating sample 1, shown in blue) or attributable to 2 or 3 potential contaminating samples. STM numbers are is in Fig. S1.

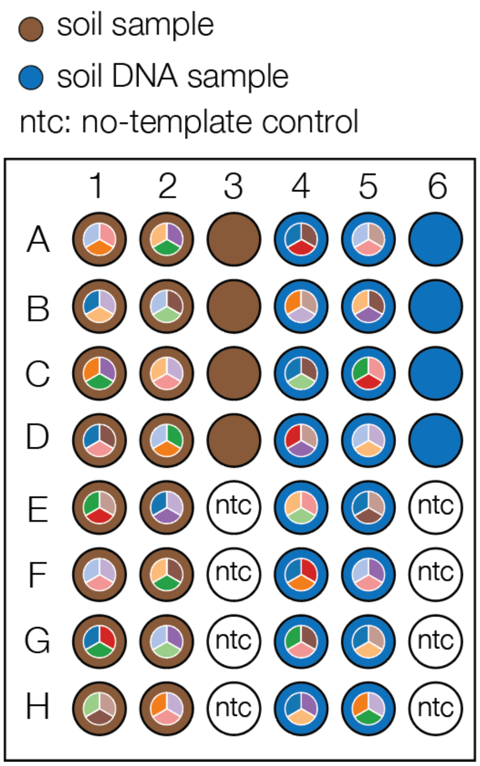

Figure S8. Schematic of the sample layout for the 'case demonstration' experiment. NTC represents no-template controls at the point of DNA extraction or first-round PCR.

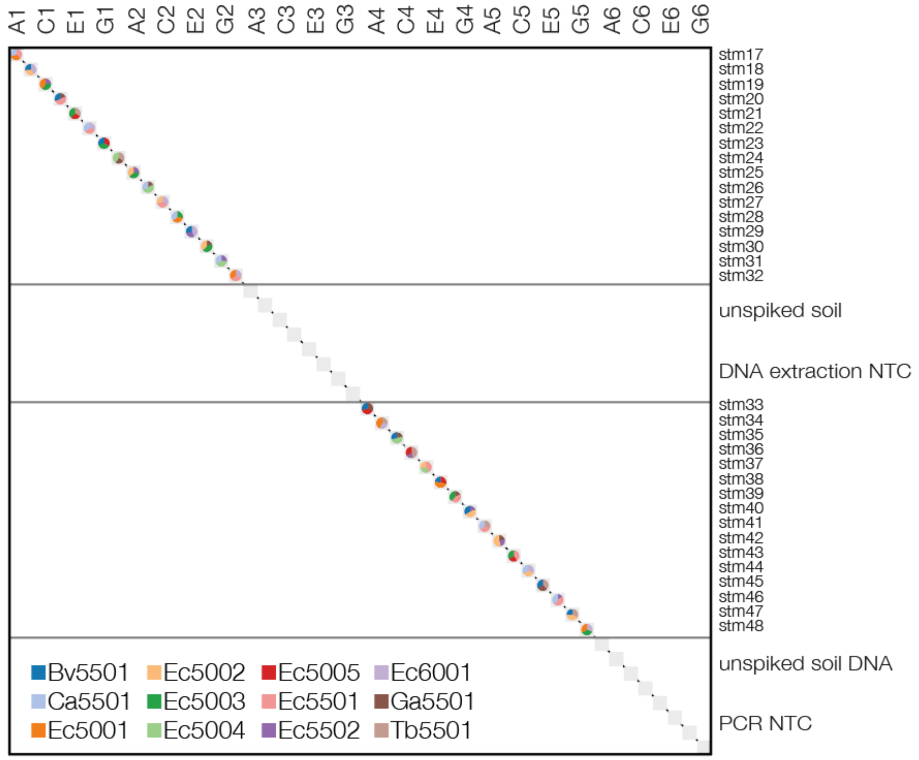

Figure S9. Map of pie charts visualizing the output of sample tracking for the 'case demonstration' experiment. Each pie chart represents the proportional abundance of the three spike-in controls in each of identified majority STMs across samples. The map is organized such that swapped samples would be located on the off-diagonal, as for Fig. 3A in the main text. Note that for the unspiked samples and no-template controls (NTCs) no majority STMs were identified. See Fig. S8 for plate layout for this experiment.

Table S1 Overview of 16S rRNA gene spike-in control sequences.

ControlID	Accession number	Reference sequence	Length (nt)	G+C content (%)
Ec5001	LC140931	*E. coli* strain ATCC 11775 (X80725, AF233451)	1,525	51.3
Ec5002	LC140932	*E. coli* strain ATCC 11775 (X80725, AF233451)	1,525	52.1
Ec5003	LC140933	*E. coli* strain ATCC 11775 (X80725, AF233451)	1,525	51.7
Ec5004	LC140934	*E. coli* strain ATCC 11775 (X80725, AF233451)	1,525	51.7
Ec5005	LC140935	*E. coli* strain ATCC 11775 (X80725, AF233451)	1,525	51.5
Ec5501	LC140936	*E. coli* strain ATCC 11775 (X80725, AF233451)	1,525	55.3
Ec5502	LC140937	*E. coli* strain ATCC 11775 (X80725, AF233451)	1,525	56.2
Ec6001	LC140938	*E. coli* strain ATCC 11775 (X80725, AF233451)	1,525	57.2
Bv5501	LC140939	*B. vulgatus* JCM 5826 (NR_112946)	1,520	55.5
Ca5501	LC140940	*C. acetobutylicum* strain ATCC 824 (X78070)	1,495	55.8
Ga5501	LC140941	*G. aurantiaca* strain T-27 (AB072735)	1,508	57.9
Tb5501	LC140942	*T. bryantii* strain DSM 1788 (NR_118718)	1,554	56.2

Adopted from Tourlousse et al., 2017 Nucleic Acids Res. 28;45(4):e23

Table S2. Composition of the 96 sample-tracking mixes (STMs) prepared in this study.

STM.identifier	STM.composition	STM.identifier	STM.composition
stm01	Bv5501,Ec6001,Ga5501	stm49	Ec5002,Ec5004,Ga5501
stm02	Ca5501,Ec5002,Ec5501	stm50	Bv5501,Ec5001,Ec5003
stm03	Ec5001,Ec5005,Tb5501	stm51	Ec5004,Ec5502,Ec6001
stm04	Ec5003,Ec5004,Ec5502	stm52	Bv5501,Ec5003,Ga5501
stm05	Bv5501,Ec5001,Ec5002	stm53	Ec5004,Ec5005,Ec5501
stm06	Ec5501,Ec6001,Tb5501	stm54	Bv5501,Ec5003,Ec6001
stm07	Ca5501,Ec5005,Ga5501	stm55	Ec5001,Ec5004,Ga5501
stm08	Bv5501,Ec5001,Ec5501	stm56	Ca5501,Ec5002,Tb5501
stm09	Ec5002,Ec5003,Tb5501	stm57	Bv5501,Ec5005,Ec5501
stm10	Ec5001,Ec5005,Ec5502	stm58	Ca5501,Ec5502,Ga5501
stm11	Bv5501,Ca5501,Ga5501	stm59	Bv5501,Ec5002,Ec5005
stm12	Ec5002,Ec5005,Ec6001	stm60	Ec5001,Ec5501,Tb5501
stm13	Ca5501,Ec5004,Ec5501	stm61	Bv5501,Ec5502,Ga5501
stm14	Bv5501,Ec5003,Ec5502	stm62	Ec5002,Ec5005,Tb5501
stm15	Ec5001,Ec5004,Tb5501	stm63	Bv5501,Ca5501,Ec5502
stm16	Ec5005,Ec5502,Ec6001	stm64	Ec5001,Ec5003,Ga5501
stm17	Ca5501,Ec5001,Ec5501	stm65	Ca5501,Ec5502,Tb5501
stm18	Bv5501,Ec5002,Ec6001	stm66	Bv5501,Ec5003,Ec5004
stm19	Ec5001,Ec5003,Ec5502	stm67	Ec5501,Ec6001,Ga5501
stm20	Bv5501,Ec5501,Ga5501	stm68	Bv5501,Ec5004,Ec5005
stm21	Ec5003,Ec5005,Tb5501	stm69	Ec5003,Ec5501,Ec6001
stm22	Ca5501,Ec5501,Ec6001	stm70	Ec5001,Ec5004,Ec5502
stm23	Bv5501,Ec5003,Ec5005	stm71	Ec5003,Ec5005,Ec6001
stm24	Ec5004,Ga5501,Tb5501	stm72	Ec5501,Ec5502,Tb5501
stm25	Ec5002,Ec5003,Ec5502	stm73	Ec5002,Ec5004,Ec6001
stm26	Ca5501,Ec5004,Ga5501	stm74	Ca5501,Ec5501,Ga5501
stm27	Ec5002,Ec5501,Ec6001	stm75	Ec5003,Ec5005,Ec5502
stm28	Ca5501,Ec5001,Ec5003	stm76	Ec5002,Ga5501,Tb5501
stm29	Bv5501,Ec5502,Ec6001	stm77	Bv5501,Ec5004,Ec5502
stm30	Ec5002,Ec5003,Ga5501	stm78	Ec5501,Ga5501,Tb5501
stm31	Ca5501,Ec5004,Ec5502	stm79	Bv5501,Ca5501,Ec5002
stm32	Ec5001,Ec5501,Ec6001	stm80	Ec5003,Ec5004,Ec6001
stm33	Bv5501,Ec5005,Ga5501	stm81	Bv5501,Ec5001,Ga5501
stm34	Ec5001,Ec6001,Tb5501	stm82	Ec5002,Ec5003,Ec6001
stm35	Bv5501,Ec5004,Ga5501	stm83	Bv5501,Ec5501,Tb5501
stm36	Ec5005,Ec5502,Tb5501	stm84	Ec5004,Ec5005,Ec6001
stm37	Ec5002,Ec5004,Ec5501	stm85	Ca5501,Ec5003,Ga5501
stm38	Bv5501,Ec5001,Ec5005	stm86	Bv5501,Ec5005,Ec6001
stm39	Ec5003,Ec5501,Ga5501	stm87	Ec5004,Ec5501,Ec5502
stm40	Bv5501,Ec5002,Ec5502	stm88	Ca5501,Ec5001,Tb5501
stm41	Ca5501,Ec5501,Tb5501	stm89	Ec5004,Ec5005,Ec5502
stm42	Ec5002,Ec5502,Ga5501	stm90	Ec5001,Ga5501,Tb5501
stm43	Ec5003,Ec5005,Ec5501	stm91	Ec5002,Ec5502,Ec6001
stm44	Ca5501,Ec5002,Ec6001	stm92	Ca5501,Ga5501,Tb5501
stm45	Bv5501,Ga5501,Tb5501	stm93	Ec5005,Ec5501,Ec6001
stm46	Ca5501,Ec5501,Ec5502	stm94	Ca5501,Ec5003,Ec5502
stm47	Bv5501,Ec5002,Tb5501	stm95	Bv5501,Ec5001,Ec6001
stm48	Ec5001,Ec5003,Ec6001	stm96	Ec5002,Ec5003,Ec5005

Table S3. Read processing statistics.

lib.ID	fastq.ID	sample.ID	demultiplexed.reads	quality-trimmed.reads	merged.reads	primer-trimmed.reads
e1-stm01	sam459_S349	validation [stm01]	185,974	173,891	170,102	168,545
e1-stm02	sam102_S257	validation [stm02]	295,535	277,485	271,923	269,312
e1-stm03	sam103_S269	validation [stm03]	166,184	156,897	153,903	152,413
e1-stm04	sam104_S263	validation [stm04]	623,602	581,170	571,985	566,693
e1-stm05	sam105_S267	validation [stm05]	471,805	448,048	439,649	435,549
e1-stm06	sam106_S266	validation [stm06]	602,638	573,432	564,091	558,859
e1-stm07	sam107_S259	validation [stm07]	630,352	592,765	578,819	573,387
e1-stm08	sam108_S270	validation [stm08]	435,409	412,989	405,754	401,922
e1-stm09	sam117_S155	validation [stm09]	50,781	47,722	46,907	46,468
e1-stm10	sam466_S351	validation [stm10]	37,187	34,929	34,150	33,803
e1-stm11	sam119_S164	validation [stm11]	22,858	21,604	21,275	21,069
e1-stm12	sam120_S158	validation [stm12]	261,218	250,094	246,180	243,959
e1-stm13	sam121_S162	validation [stm13]	331,408	316,393	311,548	308,726
e1-stm14	sam122_S161	validation [stm14]	105,329	98,497	97,068	96,192
e1-stm15	sam123_S154	validation [stm15]	100,762	95,550	93,934	93,085
e1-stm16	sam124_S165	validation [stm16]	187,218	176,836	174,138	172,488
e1-stm17	sam133_S50	validation [stm17]	1,699,101	1,607,146	1,576,765	1,562,237
e1-stm18	sam134_S47	validation [stm18]	259,466	240,864	235,479	233,280
e1-stm19	sam461_S352	validation [stm19]	51,696	48,497	47,552	47,110
e1-stm20	sam136_S53	validation [stm20]	333,746	313,049	307,556	304,702
e1-stm21	sam137_S57	validation [stm21]	1,689,960	1,601,916	1,570,898	1,556,046
e1-stm22	sam138_S56	validation [stm22]	176,758	166,364	163,585	162,039
e1-stm23	sam139_S49	validation [stm23]	192,664	179,240	175,099	173,401
e1-stm24	sam140_S60	validation [stm24]	354,824	332,212	326,047	323,120
e1-stm25	sam149_S320	validation [stm25]	274,244	258,480	254,179	251,826
e1-stm26	sam150_S317	validation [stm26]	97,033	90,558	88,901	88,081
e1-stm27	sam151_S329	validation [stm27]	44,577	41,843	41,193	40,824
e1-stm28	sam467_S355	validation [stm28]	50,335	46,945	46,049	45,589
e1-stm29	sam153_S327	validation [stm29]	110,586	100,667	98,899	97,949
e1-stm30	sam154_S326	validation [stm30]	37,405	33,803	32,411	32,083
e1-stm31	sam155_S319	validation [stm31]	82,361	76,031	74,685	73,957
e1-stm32	sam156_S330	validation [stm32]	195,887	184,978	182,107	180,416
e1-stm33	sam165_S215	validation [stm33]	242,787	226,571	223,471	221,387
e1-stm34	sam166_S212	validation [stm34]	149,023	140,659	138,850	137,565
e1-stm35	sam167_S224	validation [stm35]	90,018	84,806	83,733	82,980
e1-stm36	sam168_S218	validation [stm36]	80,067	75,728	74,798	74,103
e1-stm37	sam462_S348	validation [stm37]	251,615	237,270	231,772	229,572
e1-stm38	sam170_S221	validation [stm38]	135,173	127,058	125,482	124,235
e1-stm39	sam171_S214	validation [stm39]	122,722	113,006	111,575	110,505
e1-stm40	sam172_S225	validation [stm40]	199,667	189,848	187,530	185,725
e1-stm41	sam181_S305	validation [stm41]	1,920,367	1,830,734	1,796,880	1,780,144
e1-stm42	sam182_S302	validation [stm42]	537,555	507,276	495,834	491,136
e1-stm43	sam183_S314	validation [stm43]	140,416	131,363	128,949	127,727
e1-stm44	sam184_S308	validation [stm44]	482,668	453,196	445,512	441,391
e1-stm45	sam185_S312	validation [stm45]	425,765	400,373	392,235	388,488
e1-stm46	sam468_S358	validation [stm46]	125,012	117,325	114,902	113,887
e1-stm47	sam187_S304	validation [stm47]	153,015	143,918	140,820	139,467
e1-stm48	sam188_S315	validation [stm48]	271,137	255,317	250,654	248,256
e1-stm49	sam197_S170	validation [stm49]	467,898	446,491	439,375	435,224
e1-stm50	sam198_S167	validation [stm50]	120,375	112,552	110,866	109,845
e1-stm51	sam199_S179	validation [stm51]	110,884	104,359	102,888	101,958
e1-stm52	sam200_S173	validation [stm52]	498,935	476,538	469,855	465,579
e1-stm53	sam201_S177	validation [stm53]	245,567	232,830	229,563	227,523
e1-stm54	sam202_S176	validation [stm54]	282,660	268,208	264,608	262,087
e1-stm55	sam463_S346	validation [stm55]	462,147	438,230	429,423	425,510
e1-stm56	sam204_S180	validation [stm56]	164,500	154,257	152,094	150,624
e1-stm57	sam213_S80	validation [stm57]	201,407	189,943	185,815	184,092
e1-stm58	sam214_S77	validation [stm58]	50,422	46,607	45,569	45,123
e1-stm59	sam215_S89	validation [stm59]	257,864	245,316	240,157	237,869
e1-stm60	sam216_S83	validation [stm60]	907,786	868,932	854,544	846,976
e1-stm61	sam217_S87	validation [stm61]	226,081	212,405	208,290	206,385
e1-stm62	sam218_S86	validation [stm62]	193,097	182,671	179,300	177,649
e1-stm63	sam219_S79	validation [stm63]	951,585	902,743	883,081	874,786

e1-stm64	sam475_S334	validation [stm64]	268,961	248,407	242,748	240,436
e1-stm65	sam460_S360	validation [stm65]	234,578	223,201	219,102	217,131
e1-stm66	sam230_S197	validation [stm66]	88,994	84,787	83,750	82,990
e1-stm67	sam231_S209	validation [stm67]	70,411	67,600	66,825	66,181
e1-stm68	sam232_S203	validation [stm68]	46,218	43,311	42,841	42,418
e1-stm69	sam233_S207	validation [stm69]	72,012	68,269	67,521	66,890
e1-stm70	sam234_S206	validation [stm70]	52,330	49,573	49,048	48,560
e1-stm71	sam235_S199	validation [stm71]	66,699	62,769	61,947	61,292
e1-stm72	sam236_S210	validation [stm72]	66,552	62,805	62,121	61,535
e1-stm73	sam245_S110	validation [stm73]	98,271	91,896	90,166	89,323
e1-stm74	sam477_S337	validation [stm74]	22,745	21,244	20,819	20,613
e1-stm75	sam247_S119	validation [stm75]	45,961	42,422	41,685	41,307
e1-stm76	sam248_S113	validation [stm76]	230,673	219,900	216,219	214,287
e1-stm77	sam249_S117	validation [stm77]	271,763	257,165	252,660	250,369
e1-stm78	sam250_S116	validation [stm78]	163,301	155,083	152,626	151,196
e1-stm79	sam251_S109	validation [stm79]	226,412	214,332	210,116	208,162
e1-stm80	sam252_S120	validation [stm80]	171,587	161,558	158,834	157,353
e1-stm81	sam261_S5	validation [stm81]	2,109,141	2,008,732	1,971,168	1,952,690
e1-stm82	sam262_S2	validation [stm82]	325,030	302,480	296,233	293,436
e1-stm83	sam465_S354	validation [stm83]	60,659	57,554	56,495	55,942
e1-stm84	sam264_S8	validation [stm84]	223,916	208,842	205,236	203,246
e1-stm85	sam265_S12	validation [stm85]	21,918	20,030	19,664	19,482
e1-stm86	sam266_S11	validation [stm86]	372,907	353,038	346,912	343,672
e1-stm87	sam267_S4	validation [stm87]	303,023	282,674	276,644	273,970
e1-stm88	sam268_S15	validation [stm88]	832,596	793,990	780,255	773,213
e1-stm89	sam277_S245	validation [stm89]	343,063	321,594	315,574	312,533
e1-stm90	sam278_S242	validation [stm90]	1,950,994	1,858,259	1,825,690	1,808,737
e1-stm91	sam279_S254	validation [stm91]	143,083	134,831	132,773	131,531
e1-stm92	sam478_S333	validation [stm92]	180,751	170,672	166,911	165,390
e1-stm93	sam281_S252	validation [stm93]	164,247	153,284	150,945	149,504
e1-stm94	sam282_S251	validation [stm94]	169,406	160,937	158,646	157,198
e1-stm95	sam283_S244	validation [stm95]	1,601,192	1,534,878	1,511,035	1,498,831
e1-stm96	sam284_S255	validation [stm96]	437,955	415,540	409,247	405,466
e2-2101	sam397_S292	swap [sam01.stm01]	368,242	348,185	342,208	339,522
e2-2102	sam398_S288	swap [sam02.stm02]	286,069	268,995	264,181	262,124
e2-2103	sam399_S286	swap [sam03.stm03]	566,139	533,869	525,024	520,995
e2-2104	sam396_S300	swap [sam04.stm04]	623,310	591,256	582,157	577,649
e2-2105	sam401_S294	swap [sam05.stm05]	804,709	765,811	754,486	748,498
e2-2106	sam402_S291	swap [sam06.stm06]	86,887	80,171	78,792	78,173
e2-2107	sam403_S295	swap [sam07.stm07]	531,943	503,118	494,330	490,432
e2-2108	sam404_S298	swap [sam08.stm08]	374,597	354,645	348,774	346,111
e2-2109	sam479_S331	swap [sam09.stm09]	1,192,439	1,119,076	1,099,343	1,090,998
e2-2110	sam414_S33	swap [sam10.stm10]	103,790	95,010	93,426	92,711
e2-2111	sam415_S31	swap [sam11.stm11]	147,597	134,000	131,881	130,861
e2-2112	sam412_S45	swap [sam12.stm12]	125,685	119,191	117,418	116,530
e2-2113	sam417_S39	swap [sam13.stm13]	63,668	59,424	58,602	58,131
e2-2114	sam418_S36	swap [sam14.stm14]	77,569	72,040	70,859	70,299
e2-2115	sam419_S40	swap [sam15.stm15]	96,870	89,727	88,345	87,649
e2-2116	sam482_S336	swap [sam16.stm16]	192,558	175,941	172,631	171,280
e2-2201	sam430_S93	contamination [100%sewage.0%soil] rep1	886,680	834,578	817,355	811,086
e2-2202	sam446_S123	contamination [100%sewage.0%soil] rep2	1,021,882	963,022	943,499	936,316
e2-2203	sam429_S97	contamination [0%sewage.100%soil] rep1	476,241	448,170	440,116	436,677
e2-2204	sam445_S127	contamination [0%sewage.100%soil] rep2	144,630	134,175	131,886	130,848
e2-2205	sam476_S345	contamination [50%sewage.50%soil] rep1	429,665	407,995	400,637	397,552
e2-2206	sam447_S121	contamination [50%sewage.50%soil] rep2	73,404	69,029	67,904	67,387
e2-2207	sam428_S105	contamination [90%sewage.10%soil] rep1	527,372	502,122	493,109	489,224
e2-2208	sam484_S343	contamination [90%sewage.10%soil] rep2	788,137	747,907	733,393	727,776
e2-2209	sam433_S99	contamination [99%sewage.1%soil] rep1	445,773	424,343	416,665	413,429
e2-2210	sam481_S339	contamination [99%sewage.1%soil] rep2	826,330	782,841	769,051	762,962
e2-2211	sam483_S340	contamination [99.5%sewage.0.5%soil] rep1	579,948	550,322	539,439	534,878
e2-2212	sam450_S126	contamination [99.5%sewage.0.5%soil] rep2	248,947	235,359	230,920	229,148
e2-2213	sam435_S100	contamination [99.9%sewage.0.1%soil] rep1	639,479	604,884	593,405	588,728
e2-2214	sam451_S130	contamination [99.9%sewage.0.1%soil] rep2	678,062	642,931	631,656	626,684
e3-A1	sam301_S22	[well A1] soil.stm17	194,202	181,076	177,781	176,241
e3-A2	sam317_S232	[well A2] soil.stm25	59,324	54,655	53,661	53,196
e3-A3	sam334_S183	[well A3] unspiked.soil.A3	60,473	55,832	54,558	54,042
e3-A4	sam349_S142	[well A4] soilDNA.stm33	8,278	7,398	7,263	7,206
e3-A5	sam365_S277	[well A5] soilDNA.stm41	82,909	78,636	77,253	76,651
e3-A6	sam382_S63	[well A6] unspiked.soil.DNA.A6	122,460	115,894	113,902	112,997
e3-B1	sam302_S18	[well B1] soil.stm18	458,535	431,417	423,288	419,631

e3-B2	sam318_S228	[well B2] soil.stm26	168,427	159,318	156,162	154,865
e3-B3	sam335_S181	[well B3] unspiked.soil.B3	1,084,332	1,028,854	1,011,004	1,003,139
e3-B4	sam350_S138	[well B4] soilDNA.stm34	51,073	47,979	47,105	46,762
e3-B5	sam366_S273	[well B5] soilDNA.stm42	52,365	49,516	48,575	48,188
e3-B6	sam383_S61	[well B6] unspiked.soil.DNA.B6	110,271	104,850	103,307	102,515
e3-C1	sam303_S16	[well C1] soil.stm19	400,738	377,545	370,996	367,801
e3-C2	sam319_S226	[well C2] soil.stm27	80,212	75,695	74,412	73,784
e3-C3	sam332_S195	[well C3] unspiked.soil.C3	346,733	320,676	313,962	311,270
e3-C4	sam351_S136	[well C4] soilDNA.stm35	32,070	29,915	29,469	29,246
e3-C5	sam367_S271	[well C5] soilDNA.stm43	16,230	15,087	14,797	14,705
e3-C6	sam380_S75	[well C6] unspiked.soil.DNA.C6	44,805	41,978	41,239	40,903
e3-D1	sam300_S30	[well D1] soil.stm20	134,110	122,218	120,298	119,256
e3-D2	sam316_S240	[well D2] soil.stm28	67,215	62,475	61,546	61,079
e3-D3	sam333_S187	[well D3] unspiked.soil.C4	85,850	80,354	78,911	78,324
e3-D4	sam348_S150	[well D4] soilDNA.stm36	18,818	17,467	17,215	17,102
e3-D5	sam364_S285	[well D5] soilDNA.stm44	23,037	21,709	21,384	21,212
e3-D6	sam381_S67	[well D6] unspiked.soil.DNA.D6	39,696	37,232	36,628	36,355
e3-E1	sam305_S24	[well E1] soil.stm21	205,201	189,819	186,833	185,281
e3-E2	sam321_S234	[well E2] soil.stm29	92,217	85,990	84,730	84,028
e3-E3	sam337_S189	[well E3] DNAextraction.NTC.E3	2,616	1,075	1,048	1,044
e3-E4	sam353_S144	[well E4] soilDNA.stm37	68,957	65,676	64,756	64,258
e3-E5	sam369_S279	[well E5] soilDNA.stm45	4,910	4,560	4,473	4,432
e3-E6	sam385_S69	[well E6] PCR.NTC.E6	2,802	61	34	34
e3-F1	sam306_S21	[well F1] soil.stm22	119,523	110,266	108,326	107,400
e3-F2	sam322_S231	[well F2] soil.stm30	133,794	125,660	123,463	122,462
e3-F3	sam338_S186	[well F3] DNAextraction.NTC.F3	1,513	466	448	446
e3-F4	sam354_S141	[well F4] soilDNA.stm38	85,535	80,954	79,528	78,932
e3-F5	sam370_S276	[well F5] soilDNA.stm46	98,039	92,851	91,140	90,481
e3-F6	sam386_S66	[well F6] PCR.NTC.F6	3,157	214	175	172
e3-G1	sam307_S25	[well G1] soil.stm23	151,780	140,277	137,933	136,761
e3-G2	sam323_S235	[well G2] soil.stm31	185,914	174,479	171,511	170,061
e3-G3	sam339_S190	[well G3] DNAextraction.NTC.G3	956	72	66	66
e3-G4	sam355_S145	[well G4] soilDNA.stm39	35,002	33,003	32,503	32,252
e3-G5	sam371_S280	[well G5] soilDNA.stm47	25,780	24,184	23,795	23,616
e3-G6	sam387_S70	[well G6] PCR.NTC.G6	96	3	2	2
e3-H1	sam308_S28	[well H1] soil.stm24	177,007	161,868	159,166	157,852
e3-H2	sam324_S238	[well H2] soil.stm32	446,485	421,773	414,589	411,230
e3-H3	sam340_S193	[well H3] DNAextraction.NTC.H3	2,288	346	325	323
e3-H4	sam356_S148	[well H4] soilDNA.stm40	41,765	39,429	38,826	38,497
e3-H5	sam372_S283	[well H5] soilDNA.stm48	66,185	62,038	60,967	60,465
e3-H6	sam388_S73	[well H6] PCR.NTC.H6	4,242	576	536	533

Table S4. Read counts for STM libraries (n=96).

lib.ID	fastq.ID	Bv5501	Ca5501	Ec5001	Ec5002	Ec5003	Ec5004	Ec5005	Ec5501	Ec5502	Ec6001	Ga5501	Tb5501	Mapped reads total	Mapped reads expected	Mapped reads unexpected	purity.%
e1-stm01	sam459_S349	52,454	4	1	2	1	0	0	0	0	68,853	46,592	1	167,908	167,899	9	99.9946
e1-stm02	sam102_S257	7	85,247	0	87,939	0	2	0	95,560	3	0	4	0	268,762	268,746	16	99.9940
e1-stm03	sam103_S269	1	0	57,822	0	0	2	45,291	0	0	0	0	48,812	151,928	151,925	3	99.9980
e1-stm04	sam104_S263	0	6	2	0	210,857	214,229	1	5	140,500	1	0	0	565,601	565,586	15	99.9973
e1-stm05	sam105_S267	101,306	0	179,169	154,185	0	0	0	2	0	3	1	0	434,666	434,660	6	99.9986
e1-stm06	sam106_S266	2	1	0	1	4	0	4	208,893	2	164,867	2	182,848	556,624	556,608	16	99.9971
e1-stm07	sam107_S259	1	220,181	35	2	4	32	219,944	2	28	1	132,217	4	572,451	572,342	109	99.9810
e1-stm08	sam108_S270	95,371	0	156,220	4	4	2	1	149,517	2	2	0	1	401,124	401,108	16	99.9960
e1-stm09	sam117_S155	0	4	0	15,383	16,384	5	2	2	0	3	1	14,504	46,288	46,271	17	99.9633
e1-stm10	sam466_S351	0	0	13,942	1	0	0	12,065	1	7,725	1	0	0	33,735	33,732	3	99.9911
e1-stm11	sam119_S164	7,091	8,537	0	0	0	0	0	0	1	0	5,390	0	21,019	21,018	1	99.9952
e1-stm12	sam120_S158	0	0	0	84,196	0	0	82,843	0	0	75,922	0	0	242,961	242,961	0	100.0000
e1-stm13	sam121_S162	2	76,095	0	1	2	105,411	1	126,598	11	14	0	0	308,135	308,104	31	99.9899
e1-stm14	sam122_S161	28,052	0	1	0	40,264	0	17	16	27,635	0	0	1	95,986	95,951	35	99.9635
e1-stm15	sam123_S154	1	0	33,854	0	0	32,012	0	1	1	0	0	26,944	92,813	92,810	3	99.9968
e1-stm16	sam124_S165	0	0	1	0	2	1	65,027	1	47,783	59,039	0	0	171,854	171,849	5	99.9971
e1-stm17	sam133_S50	3	406,385	606,781	18	22	3	14	546,110	6	5	3	9	1,559,359	1,559,276	83	99.9947
e1-stm18	sam134_S47	63,854	0	0	87,507	0	3	15	27	0	81,007	0	2	232,415	232,368	47	99.9798
e1-stm19	sam461_S352	0	0	18,800	0	17,427	0	0	0	10,794	0	0	0	47,021	47,021	0	100.0000
e1-stm20	sam136_S53	88,988	0	2	2	2	0	4	139,610	1	0	75,392	0	304,001	303,990	11	99.9964
e1-stm21	sam137_S57	1	3	3	1	551,830	1	507,948	60	0	0	30	490,909	1,550,786	1,550,687	99	99.9936
e1-stm22	sam138_S56	0	51,254	0	0	0	0	0	60,528	0	49,774	0	4	161,560	161,556	4	99.9975
e1-stm23	sam139_S49	43,192	5	3	0	67,955	0	61,878	4	1	1	10	1	173,050	173,025	25	99.9856
e1-stm24	sam140_S60	2	1	16	0	2	131,600	10	1	0	11	72,683	117,547	321,873	321,830	43	99.9866
e1-stm25	sam149_S320	1	0	0	86,051	102,247	1	0	1	62,970	0	0	0	251,271	251,268	3	99.9988
e1-stm26	sam150_S317	1	31,364	0	0	0	36,739	0	0	0	2	19,764	0	87,870	87,867	3	99.9966
e1-stm27	sam151_S329	1	0	0	12,707	0	0	0	16,119	0	11,881	0	0	40,708	40,707	1	99.9975
e1-stm28	sam467_S355	0	13,098	17,196	0	15,209	0	0	0	1	0	0	0	45,504	45,503	1	99.9978
e1-stm29	sam153_S327	30,503	0	0	1	1	0	0	1	29,221	37,823	0	0	97,550	97,547	3	99.9969
e1-stm30	sam154_S326	1	0	0	11,427	14,054	0	0	0	0	0	6,530	1	32,013	32,011	2	99.9938
e1-stm31	sam155_S319	0	23,561	0	0	0	30,387	1	3	19,833	0	0	0	73,785	73,781	4	99.9946
e1-stm32	sam156_S330	0	6	64,840	2	4	0	0	66,517	0	48,555	0	1	179,925	179,912	13	99.9928
e1-stm33	sam165_S215	69,513	0	4	0	0	0	92,348	0	1	2	58,947	1	220,816	220,808	8	99.9964
e1-stm34	sam166_S212	0	0	54,205	0	0	0	1	0	1	39,044	0	43,689	136,940	136,938	2	99.9985
e1-stm35	sam167_S224	25,937	0	0	2	0	36,176	0	1	0	1	20,682	0	82,799	82,795	4	99.9952
e1-stm36	sam168_S218	0	0	0	0	0	1	26,602	0	19,679	0	0	27,577	73,859	73,858	1	99.9986
e1-stm37	sam462_S348	0	0	1	64,404	0	69,252	1	95,439	3	1	1	0	229,102	229,095	7	99.9969
e1-stm38	sam170_S221	29,724	0	51,858	9	0	0	42,368	1	0	5	0	0	123,965	123,950	15	99.9879
e1-stm39	sam171_S214	0	1	1	0	41,651	0	1	45,086	0	0	23,533	0	110,273	110,270	3	99.9973
e1-stm40	sam172_S225	54,089	0	0	76,580	0	1	2	1	54,643	0	0	0	185,316	185,312	4	99.9978
e1-stm41	sam181_S305	3	554,721	1	3	0	1	0	647,229	1	0	3	572,137	1,774,099	1,774,087	12	99.9993
e1-stm42	sam182_S302	0	1	1	215,742	8	7	0	2	154,216	1	119,983	0	489,961	489,941	20	99.9959
e1-stm43	sam183_S314	0	0	0	0	44,491	0	35,032	47,964	0	6	0	0	127,493	127,487	6	99.9953
e1-stm44	sam184_S308	5	147,644	0	155,105	0	1	3	0	0	137,155	2	0	439,915	439,904	11	99.9975
e1-stm45	sam185_S312	119,431	0	0	0	6	0	3	6	0	2	102,188	165,223	386,859	386,842	17	99.9956
e1-stm46	sam468_S358	1	37,956	0	4	0	1	0	46,861	28,856	0	0	1	113,680	113,673	7	99.9938
e1-stm47	sam187_S304	36,384	2	2	52,569	6	0	5	0	0	9	1	49,989	138,967	138,942	25	99.9820
e1-stm48	sam188_S315	0	20	92,492	0	86,509	10	1	3	8	68,471	14	7	247,535	247,472	63	99.9745
e1-stm49	sam197_S170	3	0	1	160,999	1	177,452	4	2	1	9	95,941	0	434,413	434,392	21	99.9952
e1-stm50	sam198_S167	26,374	0	43,580	0	39,649	1	0	0	1	0	0	0	109,605	109,603	2	99.9982
e1-stm51	sam199_S179	1	0	0	0	0	40,168	0	0	29,467	32,018	0	0	101,654	101,653	1	99.9990
e1-stm52	sam200_S173	136,803	0	0	3	210,486	3	0	1	0	1	117,156	29	464,482	464,445	37	99.9920
e1-stm53	sam201_S177	0	1	0	0	3	79,906	66,987	80,217	2	0	2	0	227,118	227,110	8	99.9965
e1-stm54	sam202_S176	70,665	1	0	0	104,848	3	1	3	0	85,560	0	0	261,081	261,073	8	99.9969
e1-stm55	sam463_S346	1	0	177,940	0	0	161,464	0	0	1	1	85,301	0	424,708	424,705	3	99.9993
e1-stm56	sam204_S180	0	47,242	0	50,668	0	1	0	1	0	0	0	52,092	150,004	150,002	2	99.9987
e1-stm57	sam213_S80	46,520	2	1	0	0	0	63,735	73,451	2	0	3	1	183,715	183,706	9	99.9951
e1-stm58	sam214_S77	0	18,583	1	0	1	1	0	1	14,379	0	12,046	1	45,013	45,008	5	99.9889
e1-stm59	sam215_S89	70,252	0	0	87,291	0	0	79,827	1	2	0	1	3	237,377	237,370	7	99.9971
e1-stm60	sam216_S83	0	0	303,200	18	1	0	2	292,454	11	10	2	248,704	844,402	844,358	44	99.9948
e1-stm61	sam217_S87	72,189	1	4	0	1	1	1	1	73,143	0	60,544	2	205,887	205,876	11	99.9947

e1-stm62	sam218_S86	0	0	2	63,667	0	0	57,200	0	0	1	0	56,187	177,057	177,054	3	99.9983
e1-stm63	sam219_S79	264,958	339,587	5	2	0	2	1	3	268,410	3	0	0	872,971	872,955	16	99.9982
e1-stm64	sam475_S334	0	0	98,667	0	91,210	0	1	1	1	1	50,093	2	239,976	239,970	6	99.9975
e1-stm65	sam460_S360	2	70,171	1	9	18	1	0	0	61,558	1	5	84,577	216,343	216,306	37	99.9829
e1-stm66	sam230_S197	19,894	0	0	0	30,974	31,933	0	0	0	0	0	0	82,801	82,801	0	100.0000
e1-stm67	sam231_S209	3	0	0	0	0	0	1	29,385	2	21,935	14,599	0	65,925	65,919	6	99.9909
e1-stm68	sam232_S203	11,154	0	1	0	0	16,686	14,466	0	0	0	0	0	42,307	42,306	1	99.9976
e1-stm69	sam233_S207	0	1	0	0	23,370	0	0	24,119	0	19,206	0	0	66,696	66,695	1	99.9985
e1-stm70	sam234_S206	0	0	19,188	0	0	17,954	0	1	11,330	0	2	0	48,475	48,472	3	99.9938
e1-stm71	sam235_S199	1	1	0	0	22,136	0	20,534	0	0	18,416	0	1	61,089	61,086	3	99.9951
e1-stm72	sam236_S210	0	0	0	0	0	0	0	25,162	15,348	0	0	20,811	61,321	61,321	0	100.0000
e1-stm73	sam245_S110	2	0	0	30,409	0	32,542	0	2	1	26,050	0	1	89,007	89,001	6	99.9933
e1-stm74	sam477_S337	0	6,715	1	0	1	0	0	9,069	0	0	4,756	0	20,542	20,540	2	99.9903
e1-stm75	sam247_S119	0	0	0	0	16,120	20	13,198	1	11,879	5	0	0	41,223	41,197	26	99.9369
e1-stm76	sam248_S113	0	0	0	82,616	0	0	0	1	0	0	49,134	81,770	213,521	213,520	1	99.9995
e1-stm77	sam249_S117	70,783	0	0	0	1	108,496	0	1	70,514	0	1	0	249,796	249,793	3	99.9988
e1-stm78	sam250_S116	1	2	1	1	0	0	0	63,843	0	0	33,324	53,471	150,643	150,638	5	99.9967
e1-stm79	sam251_S109	59,122	70,669	1	77,863	0	1	0	0	1	0	2	0	207,659	207,654	5	99.9976
e1-stm80	sam252_S120	0	0	0	0	55,652	55,561	0	0	0	45,705	0	0	156,918	156,918	0	100.0000
e1-stm81	sam261_S5	560,202	2	914,756	0	1	1	0	1	8	12	473,486	0	1,948,469	1,948,444	25	99.9987
e1-stm82	sam262_S2	1	0	4	99,587	105,288	0	0	0	1	87,532	1	0	292,414	292,407	7	99.9976
e1-stm83	sam465_S354	13,247	0	0	0	0	0	0	25,624	0	1	1	16,884	55,757	55,755	2	99.9964
e1-stm84	sam264_S8	2	0	0	2	0	75,343	65,943	3	1	61,181	0	1	202,476	202,467	9	99.9956
e1-stm85	sam265_S12	0	7,279	0	0	7,662	1	0	1	0	0	4,503	0	19,446	19,444	2	99.9897
e1-stm86	sam266_S11	99,830	0	0	1	0	1	123,614	0	0	118,942	0	0	342,388	342,386	2	99.9994
e1-stm87	sam267_S4	8	2	2	1	1	99,395	1	107,871	66,132	2	1	0	273,416	273,398	18	99.9934
e1-stm88	sam268_S15	1	197,657	308,957	1	0	0	0	1	0	0	0	263,899	770,516	770,513	3	99.9996
e1-stm89	sam277_S245	1	0	2	0	0	130,085	105,769	1	76,062	2	2	3	311,927	311,916	11	99.9965
e1-stm90	sam278_S242	4	1	777,093	18	0	1	0	0	17	18	406,867	618,582	1,802,601	1,802,542	59	99.9967
e1-stm91	sam279_S254	1	0	0	49,278	0	0	0	1	38,579	43,217	0	0	131,076	131,074	2	99.9985
e1-stm92	sam478_S333	0	52,826	1	0	0	0	0	0	0	0	40,427	71,394	164,648	164,647	1	99.9994
e1-stm93	sam281_S252	4	0	4	1	0	3	45,420	60,432	0	43,059	1	1	148,925	148,911	14	99.9906
e1-stm94	sam282_S251	0	51,532	1	0	63,173	0	2	1	42,161	1	0	3	156,874	156,866	8	99.9949
e1-stm95	sam283_S244	370,572	3	619,923	1	0	0	3	0	0	505,014	0	1	1,495,517	1,495,509	8	99.9995
e1-stm96	sam284_S255	1	0	3	130,533	147,097	0	127,086	3	0	3	0	0	404,726	404,716	10	99.9975

Table S5. Read counts for the 'sample swapping' experiment.

lib.ID	fastq.ID	Bv5501	Ca5501	Ec5001	Ec5002	Ec5003	Ec5004	Ec5005	Ec5501	Ec5502	Ec6001	Ga5501	Tb5501	environmental.OTUs	%.spike-in.reads
e2-2101	sam397_S292	154	0	0	0	0	0	0	0	0	188	131	0	335,454	0.1408
e2-2102	sam398_S288	1	0	152	0	0	0	66	0	56	0	0	0	259,137	0.1060
e2-2103	sam399_S286	0	0	307	0	0	0	222	0	0	0	0	196	515,518	0.1404
e2-2104	sam396_S300	0	0	0	35	0	0	18	0	0	27	0	0	571,759	0.0140
e2-2105	sam401_S294	195	0	396	266	0	0	0	0	0	0	0	0	740,048	0.1157
e2-2106	sam402_S291	18	0	0	0	35	0	0	0	15	0	0	0	77,265	0.0879
e2-2107	sam403_S295	0	133	0	0	0	0	148	0	0	0	50	0	484,906	0.0682
e2-2108	sam404_S298	1	0	0	0	0	0	62	0	58	155	0	0	342,329	0.0806
e2-2109	sam479_S331	1	0	0	387	458	0	0	0	0	0	0	294	1,079,024	0.1055
e2-2110	sam414_S33	0	21	0	27	0	0	0	26	0	0	0	0	91,624	0.0807
e2-2111	sam415_S31	46	117	0	0	0	0	0	0	0	0	42	0	129,294	0.1583
e2-2112	sam412_S45	0	0	0	0	22	39	0	0	16	1	0	0	115,193	0.0677
e2-2113	sam417_S39	0	14	0	0	0	7	0	15	0	0	0	0	57,487	0.0626
e2-2114	sam418_S36	0	0	0	0	0	0	0	20	0	16	0	21	69,447	0.0820
e2-2115	sam419_S40	0	0	66	0	0	51	0	0	0	0	0	48	86,449	0.1905
e2-2116	sam482_S336	4	0	3	0	0	0	0	14	0	0	0	0	169,246	0.0124

Table S6. Summary output for the 'sample swapping' experiment.

sample.ID	expected.STM	total.reads	control.reads	majority.STM	majority.STM.reads	minority.STM	minority.STM.reads	unassigned.spikes.reads	unassigned.spikes.reads(%total)		QC result
swap [sam01.stm01]	stm01	335,927	473	stm01	473	nd	0	0	0.000000	✓	
swap [sam02.stm02]	stm02	259,412	275	stm10	274	nd	0	1	0.000385	✗	swapped
swap [sam03.stm03]	stm03	516,243	725	stm03	725	nd	0	0	0.000000	✓	
swap [sam04.stm04]	stm04	571,839	80	stm12	80	nd	0	0	0.000000	✗	swapped
swap [sam05.stm05]	stm05	740,905	857	stm05	857	nd	0	0	0.000000	✓	
swap [sam06.stm06]	stm06	77,333	68	stm14	68	nd	0	0	0.000000	✗	swapped
swap [sam07.stm07]	stm07	485,237	331	stm07	331	nd	0	0	0.000000	✓	
swap [sam08.stm08]	stm08	342,605	276	stm16	275	nd	0	1	0.000292	✗	swapped
swap [sam09.stm09]	stm09	1,080,164	1,140	stm09	1139	nd	0	1	0.000093	✓	
swap [sam10.stm10]	stm10	91,698	74	stm02	74	nd	0	0	0.000000	✗	swapped
swap [sam11.stm11]	stm11	129,499	205	stm11	205	nd	0	0	0.000000	✓	
swap [sam12.stm12]	stm12	115,271	78	stm04	77	nd	0	1	0.000868	✗	swapped
swap [sam13.stm13]	stm13	57,523	36	stm13	36	nd	0	0	0.000000	✓	
swap [sam14.stm14]	stm14	69,504	57	stm06	57	nd	0	0	0.000000	✗	swapped
swap [sam15.stm15]	stm15	86,614	165	stm15	165	nd	0	0	0.000000	✓	
swap [sam16.stm16]	stm16	169,267	21	stm08	21	nd	0	0	0.000000	✗	swapped

Table S7. Read counts for the 'sample cross-contamination' experiment.

lib.ID	fastq.ID	Bv5501	Ca5501	Ec5001	Ec5002	Ec5003	Ec5004	Ec5005	Ec5501	Ec5502	Ec6001	Ga5501	Tb5501	environmental.OTUs	%.spike-in.reads
e2-2201	sam430_S93	1	776	704	0	0	0	0	0	0	0	0	511	790,635	0.2513
e2-2202	sam446_S123	1	823	814	0	0	0	0	0	0	0	0	540	912,257	0.2382
e2-2203	sam429_S97	0	0	0	0	0	5,630	3,791	0	2,289	0	0	0	420,461	2.7096
e2-2204	sam445_S127	0	0	0	0	0	1,531	980	0	542	0	0	0	126,512	2.3563
e2-2205	sam476_S345	1	353	346	0	0	438	279	0	206	1	0	261	388,948	0.4823
e2-2206	sam447_S121	0	61	65	0	0	72	59	0	33	0	0	36	65,906	0.4922
e2-2207	sam428_S105	1	374	472	0	1	63	30	1	31	0	0	342	476,378	0.2753
e2-2208	sam484_S343	0	608	666	0	0	91	63	1	62	0	0	484	708,698	0.2779
e2-2209	sam433_S99	0	328	371	0	0	4	2	0	1	0	0	294	402,386	0.2479
e2-2210	sam481_S339	0	753	630	0	0	6	3	0	1	0	0	505	742,300	0.2550
e2-2211	sam483_S340	0	466	447	0	0	3	3	0	1	0	0	342	520,499	0.2419
e2-2212	sam450_S126	1	193	238	0	0	1	0	0	0	0	0	139	223,117	0.2557
e2-2213	sam435_S100	0	535	490	0	0	0	0	0	0	0	0	376	573,291	0.2438
e2-2214	sam451_S130	0	525	495	0	0	0	0	0	1	0	0	393	610,127	0.2312

Table S8. Summary output for the 'sample cross-contamination' experiment.

sample.ID	expected.STM	admixture.proportion.expected(%)	total.reads	control.reads	stm.major.mixID	stm.major.mixID.reads	stm.minor.mixID	stm.minor.mixID.reads	estimated.carry-over(%)	unassigned.spikes.reads	unassigned.spikes.reads(%total)
contamination [100%sewage.0%soil] rep1	stm88	0	792,627	1,992	stm88	1,991	nd	0	0.0000	1	0.000126
contamination [100%sewage.0%soil] rep2	stm88	0	914,435	2,178	stm88	2,177	nd	0	0.0000	1	0.000109
contamination [0%sewage.100%soil] rep1	stm89	100	432,171	11,710	stm89	11,710	nd	0	0.0000	0	0.000000
contamination [0%sewage.100%soil] rep2	stm89	100	129,565	3,053	stm89	3,053	nd	0	0.0000	0	0.000000
contamination [50%sewage.50%soil] rep1	stm88+minority stm89	50	390,833	1,885	stm88	960	stm89	923	49.0175	2	0.000512
contamination [50%sewage.50%soil] rep2	stm88+minority stm89	50	66,232	326	stm89	164	stm88	162	49.6933	0	0.000000
contamination [90%sewage.10%soil] rep1	stm88+minority stm89	10	477,693	1,315	stm88	1,188	stm89	124	9.4512	3	0.000628
contamination [90%sewage.10%soil] rep2	stm88+minority stm89	10	710,673	1,975	stm88	1,758	stm89	216	10.9422	1	0.000141
contamination [99%sewage.1%soil] rep1	stm88+minority stm89	1	403,386	1,000	stm88	993	stm89	7	0.7000	0	0.000000
contamination [99%sewage.1%soil] rep2	stm88+minority stm89	1	744,198	1,898	stm88	1,888	stm89	10	0.5269	0	0.000000
contamination [99.5%sewage.0.5%soil] rep1	stm88+minority stm89	0.5	521,761	1,262	stm88	1,255	stm89	7	0.5547	0	0.000000
contamination [99.5%sewage.0.5%soil] rep2	stm88+minority stm89	0.5	223,689	572	stm88	570	nd	0	0.0000	2	0.000894
contamination [99.9%sewage.0.1%soil] rep1	stm88+minority stm89	0.1	574,692	1,401	stm88	1,401	nd	0	0.0000	0	0.000000
contamination [99.9%sewage.0.1%soil] rep2	stm88+minority stm89	0.1	611,541	1,414	stm88	1,413	nd	0	0.0000	1	0.000164

Table S9. Read counts for the 'case demonstration'.

lib.ID	fastq.ID	Bv5501	Ca5501	Ec5001	Ec5002	Ec5003	Ec5004	Ec5005	Ec5501	Ec5502	Ec6001	Ga5501	Tb5501	environmental.OTUs	%.spike-in.reads
e3-A1	sam301_S22	0	1,757	1,921	0	0	0	0	2,216	0	0	0	0	167,762	3.3941
e3-B1	sam302_S18	2,488	0	0	3,229	0	0	0	0	0	3,951	1	0	404,486	2.3346
e3-C1	sam303_S16	0	0	4,699	0	4,182	0	0	0	2,824	0	0	0	350,980	3.2273
e3-D1	sam300_S30	598	0	1	0	0	0	0	980	0	0	373	1	115,725	1.6596
e3-E1	sam305_S24	0	0	0	0	900	0	711	0	0	0	0	804	180,023	1.3237
e3-F1	sam306_S21	0	706	0	0	0	1	0	708	0	642	0	0	103,823	1.9428
e3-G1	sam307_S25	841	0	0	0	1,105	4	1,000	0	0	0	0	0	131,938	2.1870
e3-H1	sam308_S28	0	0	0	0	0	1,958	0	0	0	0	1,069	1,687	151,012	3.0271
e3-A2	sam317_S232	0	0	0	303	310	0	0	0	215	0	0	0	51,530	1.5814
e3-B2	sam318_S228	0	1,309	0	0	0	1,943	0	0	0	0	711	0	148,786	2.5945
e3-C2	sam319_S226	0	0	0	747	0	0	0	989	0	895	0	0	69,950	3.6249
e3-D2	sam316_S240	1	490	471	0	395	0	2	0	0	0	0	0	58,406	2.2739
e3-E2	sam321_S234	264	0	0	0	0	0	0	0	254	406	0	0	81,596	1.1197
e3-F2	sam322_S231	0	0	0	535	622	0	0	0	0	1	275	0	118,809	1.1918
e3-G2	sam323_S235	0	843	0	0	0	1,036	0	0	676	0	7	15	165,133	1.5366
e3-H2	sam324_S238	0	0	6,262	0	0	37	0	6,513	0	5,617	21	31	387,235	4.5552
e3-A3	sam334_S183	0	1	0	0	0	0	1	0	0	0	1	0	53,053	0.0057
e3-B3	sam335_S181	0	0	0	0	0	0	0	0	0	0	0	0	986,808	0.0000
e3-C3	sam332_S195	0	0	0	1	0	0	0	0	0	0	0	0	305,755	0.0003
e3-D3	sam333_S187	0	0	0	0	0	0	0	0	0	0	0	0	77,134	0.0000
e3-E3	sam337_S189	0	0	0	0	0	0	0	0	0	0	0	0	1,037	0.0000
e3-F3	sam338_S186	0	0	0	0	0	0	0	0	0	0	0	0	443	0.0000
e3-G3	sam339_S190	0	0	0	0	0	0	0	0	0	1	0	0	64	1.5385
e3-H3	sam340_S193	0	0	0	0	0	0	0	0	0	0	0	0	321	0.0000
e3-A4	sam349_S142	18	0	0	0	0	0	24	0	0	0	21	0	7,081	0.8819
e3-B4	sam350_S138	0	0	531	0	0	0	0	0	0	402	0	325	45,029	2.7178
e3-C4	sam351_S136	150	0	0	0	0	249	0	0	0	0	122	0	28,445	1.7987
e3-D4	sam348_S150	0	0	0	0	0	0	112	0	65	0	0	159	16,605	1.9834
e3-E4	sam353_S144	0	0	0	243	0	290	0	384	0	0	0	0	62,715	1.4411
e3-F4	sam354_S141	295	0	605	0	0	0	390	0	0	0	0	0	76,773	1.6525
e3-G4	sam355_S145	0	0	0	0	107	0	0	132	0	0	52	0	31,590	0.9128
e3-H4	sam356_S148	180	0	0	257	0	0	0	0	113	0	0	0	37,528	1.4444
e3-A5	sam365_S277	0	618	0	0	0	0	0	624	0	0	0	523	74,066	2.3275
e3-B5	sam366_S273	0	0	0	371	0	0	0	0	169	0	146	0	46,934	1.4406
e3-C5	sam367_S271	0	0	0	0	117	0	90	145	0	0	0	0	14,205	2.4181
e3-D5	sam364_S285	0	161	0	163	0	0	0	0	0	158	0	0	20,499	2.2973
e3-E5	sam369_S279	21	0	0	0	0	0	0	0	0	0	20	25	4,328	1.5020
e3-F5	sam370_S276	0	737	0	0	0	0	0	880	283	0	0	0	87,554	2.1240
e3-G5	sam371_S280	111	0	0	178	0	0	0	0	0	0	0	151	22,897	1.8854
e3-H5	sam372_S283	0	0	427	0	440	0	0	0	0	416	0	0	58,422	2.1489
e3-A6	sam382_S63	0	0	0	0	0	0	0	0	0	0	0	0	111,810	0.0000
e3-B6	sam383_S61	0	0	0	0	0	0	0	0	0	0	0	0	101,434	0.0000
e3-C6	sam380_S75	0	0	0	1	0	0	0	0	0	0	0	0	40,417	0.0025
e3-D6	sam381_S67	0	0	0	0	0	0	0	0	0	0	0	0	35,979	0.0000
e3-E6	sam385_S69	0	0	0	0	0	0	0	0	0	0	0	0	31	0.0000
e3-F6	sam386_S66	0	0	0	0	0	0	0	0	0	0	0	0	167	0.0000
e3-G6	sam387_S70	0	0	0	0	0	0	0	0	0	0	0	0	2	0.0000
e3-H6	sam388_S73	0	0	0	0	0	0	0	0	0	0	0	0	527	0.0000

Table S10. Summary output for the 'case demonstration'.

sample.ID	expected.STM	total.reads	control.reads	stm.major.mixID	stm.major.mixID.reads	sample.swab QC	stm.minor.mixID	sample.cross-contamination.QC	potential.cross-contaminant	distinguishing controls	cross.comtamination.reads	estimated.carry-over (%)
[well A1] soil.stm17	stm17	173,656	5,894	stm17	5,894	✓	nd	✓				
[well B1] soil.stm18	stm18	414,155	9,669	stm18	9,668	✓	nd	✓				
[well C1] soil.stm19	stm19	362,685	11,705	stm19	11,705	✓	nd	✓				
[well D1] soil.stm20	stm20	117,678	1,953	stm20	1,951	✓	stm45	✗	E5	1	1	0.10
[well E1] soil.stm21	stm21	182,438	2,415	stm21	2,415	✓	nd	✓				
[well F1] soil.stm22	stm22	105,880	2,057	stm22	2,056	✓	nd	✓				
[well G1] soil.stm23	stm23	134,888	2,950	stm23	2,946	✓	nd	✓				
[well H1] soil.stm24	stm24	155,726	4,714	stm24	4,714	✓	nd	✓				
[well A2] soil.stm25	stm25	52,358	828	stm25	828	✓	nd	✓				
[well B2] soil.stm26	stm26	152,749	3,963	stm26	3,963	✓	nd	✓				
[well C2] soil.stm27	stm27	72,581	2,631	stm27	2,631	✓	nd	✓				
[well D2] soil.stm28	stm28	59,765	1,359	stm28	1,356	✓	stm23	✗	G1	2	3	0.31
							stm38		F4	2	3	0.34
[well E2] soil.stm29	stm29	82,520	924	stm29	924	✓	nd	✓				
[well F2] soil.stm30	stm30	120,242	1,433	stm30	1,432	✓	nd	✓				
[well G2] soil.stm31	stm31	167,710	2,577	stm31	2,555	✓	stm24	✗	H1	2	22	1.43
							stm26		B2	2	22	1.02
[well H2] soil.stm32	stm32	405,716	18,481	stm32	18,392	✓	stm24	✗	H1	3	89	0.48
							stm34		B4	2	52	0.47
[well A3] unspiked.soil.A3		77,134	0	nd	na	na	nd	✓				
[well B3] unspiked.soil.B3		53,056	3	nd	na	na	nd	✓				
[well C3] unspiked.soil.C3		986,808	0	nd	na	na	nd	✓				
[well D3] unspiked.soil.C4		305,756	1	nd	na	na	nd	✓				
[well E3] DNAextraction.NTC.E3		1,037	0	nd	na	na	nd	✓				
[well F3] DNAextraction.NTC.F3		443	0	nd	na	na	nd	✓				
[well G3] DNAextraction.NTC.G3		65	1	nd	na	na	nd	✓				
[well H3] DNAextraction.NTC.H3		321	0	nd	na	na	nd	✓				
[well A4] soilDNA.stm33	stm33	7,144	63	stm33	63	✓	nd	✓				
[well B4] soilDNA.stm34	stm34	46,287	1,258	stm34	1,258	✓	nd	✓				
[well C4] soilDNA.stm35	stm35	28,966	521	stm35	521	✓	nd	✓				
[well D4] soilDNA.stm36	stm36	16,941	336	stm36	336	✓	nd	✓				
[well E4] soilDNA.stm37	stm37	63,632	917	stm37	917	✓	nd	✓				
[well F4] soilDNA.stm38	stm38	78,063	1,290	stm38	1,290	✓	nd	✓				
[well G4] soilDNA.stm39	stm39	31,881	291	stm39	291	✓	nd	✓				
[well H4] soilDNA.stm40	stm40	38,078	550	stm40	550	✓	nd	✓				
[well A5] soilDNA.stm41	stm41	75,831	1,765	stm41	1,765	✓	nd	✓				
[well B5] soilDNA.stm42	stm42	47,620	686	stm42	686	✓	nd	✓				
[well C5] soilDNA.stm43	stm43	14,557	352	stm43	352	✓	nd	✓				
[well D5] soilDNA.stm44	stm44	20,981	482	stm44	482	✓	nd	✓				
[well E5] soilDNA.stm45	stm45	4,394	66	stm45	66	✓	nd	✓				
[well F5] soilDNA.stm46	stm46	89,454	1,900	stm46	1,900	✓	nd	✓				
[well G5] soilDNA.stm47	stm47	23,337	440	stm47	440	✓	nd	✓				
[well H5] soilDNA.stm48	stm48	59,705	1,283	stm48	1,283	✓	nd	✓				
[well A6] unspiked.soil.DNA.A6		35,979	0	nd	na	na	nd	✓				
[well B6] unspiked.soil.DNA.B6		111,810	0	nd	na	na	nd	✓				
[well C6] unspiked.soil.DNA.C6		101,434	0	nd	na	na	nd	✓				
[well D6] unspiked.soil.DNA.D6		40,418	1	nd	na	na	nd	✓				
[well E6] PCR.NTC.E6		31	0	nd	na	na	nd	✓				
[well F6] PCR.NTC.F6		167	0	nd	na	na	nd	✓				
[well G6] PCR.NTC.G6		2	0	nd	na	na	nd	✓				
[well H6] PCR.NTC.H6		527	0	nd	na	na	nd	✓				

Table S11. Summary of OTUs identified in the negative no-template controls (NTCs).

	reads								proportion (%)								
otuID	[well E3] DNAextraction.NTC.E3	[well F3] DNAextraction.NTC.F3	[well G3] DNAextraction.NTC.G3	[well H3] DNAextraction.NTC.H3	[well E6] PCR.NTC.E6	[well F6] PCR.NTC.F6	[well G6] PCR.NTC.G6	[well H6] PCR.NTC.H6	[well E3] DNAextraction.NTC.E3	[well F3] DNAextraction.NTC.F3	[well G3] DNAextraction.NTC.G3	[well H3] DNAextraction.NTC.H3	[well E6] PCR.NTC.E6	[well F6] PCR.NTC.F6	[well G6] PCR.NTC.G6	[well H6] PCR.NTC.H6	taxonomy
OTU.2770uparse	831	349	30	242	0	0	0	0	80.1	78.8	46.2	75.4	0.0	0.0	0.0	0.0	k:Bacteria p:Proteobacteria c:Alphaproteobacteria o:Rhizobiales f:Methylobacteriaceae g:Methylobacterium
OTU.384uparse	114	49	2	24	14	101	0	350	11.0	11.1	3.1	7.5	45.2	60.5	0.0	66.4	k:Bacteria p:Proteobacteria c:Betaproteobacteria o:Burkholderiales f:Comamonadaceae
OTU.6532uparse	39	7	0	11	6	23	0	34	3.8	1.6	0.0	3.4	19.4	13.8	0.0	6.5	k:Bacteria p:Proteobacteria c:Alphaproteobacteria o:Sphingomonadales f:Sphingomonadaceae
OTU.1638uparse	0	8	0	0	0	0	0	75	0.0	1.8	0.0	0.0	0.0	0.0	0.0	14.2	k:Bacteria p:Proteobacteria c:Gammaproteobacteria o:Xanthomonadales f:Xanthomonadaceae g:Stenotrophomonas
OTU.2034uparse	0	2	0	4	2	14	0	30	0.0	0.5	0.0	1.2	6.5	8.4	0.0	5.7	k:Bacteria p:Cyanobacteria c:4C0d-2 o:MLE1-12
OTU.14uparse	7	1	0	2	1	0	1	32	0.7	0.2	0.0	0.6	3.2	0.0	50.0	6.1	k:Bacteria p:Proteobacteria c:Alphaproteobacteria o:Rhizobiales f:Bradyrhizobiaceae
OTU.1727uparse	0	6	0	5	0	12	0	6	0.0	1.4	0.0	1.6	0.0	7.2	0.0	1.1	k:Bacteria p:Proteobacteria c:Betaproteobacteria o:Burkholderiales
OTU.11224uparse	8	5	1	3	0	0	0	0	0.8	1.1	1.5	0.9	0.0	0.0	0.0	0.0	k:Bacteria p:Proteobacteria c:Alphaproteobacteria o:Rhizobiales
OTU.137uparse	5	0	0	1	4	1	0	0	0.5	0.0	0.0	0.3	12.9	0.6	0.0	0.0	k:Bacteria p:Proteobacteria c:Gammaproteobacteria o:Xanthomonadales f:Sinobacteraceae
OTU.14108uparse	0	0	0	3	0	7	0	0	0.0	0.0	0.0	0.9	0.0	4.2	0.0	0.0	k:Bacteria p:Proteobacteria c:Alphaproteobacteria o:Caulobacterales f:Caulobacteraceae
OTU.15919uparse	6	2	0	1	0	0	0	0	0.6	0.5	0.0	0.3	0.0	0.0	0.0	0.0	k:Bacteria p:Proteobacteria c:Alphaproteobacteria o:Rhizobiales f:Bradyrhizobiaceae g:Balneimonas
OTU.793uparse	8	0	0	0	0	0	0	0	0.8	0.0	0.0	0.0	0.0	0.0	0.0	0.0	k:Bacteria p:Actinobacteria c:Actinobacteria o:Actinomycetales f:Microbacteriaceae g:Microbacterium
OTU.3401uparse	0	0	0	3	0	4	0	0	0.0	0.0	0.0	0.9	0.0	2.4	0.0	0.0	k:Bacteria p:Proteobacteria c:Deltaproteobacteria o:Myxococcales f:0319-6G20
OTU.5972uparse	6	1	0	0	0	0	0	0	0.6	0.2	0.0	0.0	0.0	0.0	0.0	0.0	k:Bacteria p:Bacteroidetes c:[Saprospirae] o:[Saprospirales] f:Chitinophagaceae g:Sediminibacterium
OTU.6955uparse	0	0	0	4	1	0	0	0	0.0	0.0	0.0	1.2	3.2	0.0	0.0	0.0	k:Bacteria p:Bacteroidetes c:[Saprospirae] o:[Saprospirales] f:Chitinophagaceae g:Sediminibacterium
OTU.208uparse	2	3	0	0	0	0	0	0	0.2	0.7	0.0	0.0	0.0	0.0	0.0	0.0	k:Bacteria p:Proteobacteria c:Alphaproteobacteria o:Rhodobacterales f:Rhodobacteraceae g:Paracoccus s:zeaxanthinifaciens
OTU.13uparse	0	0	3	0	0	1	0	0	0.0	0.0	4.6	0.0	0.0	0.6	0.0	0.0	k:Bacteria p:Proteobacteria c:Alphaproteobacteria o:Rhizobiales f:Hyphomicrobiaceae g:Rhodoplanes
OTU.1022uparse	0	1	0	0	2	1	0	0	0.0	0.2	0.0	0.0	6.5	0.6	0.0	0.0	k:Bacteria p:Proteobacteria c:Alphaproteobacteria o:Sphingomonadales f:Sphingomonadaceae
OTU.7233uparse	0	0	0	4	0	0	0	0	0.0	0.0	0.0	1.2	0.0	0.0	0.0	0.0	k:Bacteria p:Actinobacteria c:Actinobacteria o:Actinomycetales f:Corynebacteriaceae g:Corynebacterium
OTU.52uparse	0	0	0	3	0	0	0	0	0.0	0.0	0.0	0.9	0.0	0.0	0.0	0.0	k:Bacteria p:Proteobacteria c:Gammaproteobacteria o:Enterobacteriales f:Enterobacteriaceae
OTU.30uparse	0	0	2	0	0	0	0	0	0.0	0.0	3.1	0.0	0.0	0.0	0.0	0.0	k:Bacteria p:Acidobacteria c:DA052 o:Ellin6513
OTU.86uparse	0	0	2	0	0	0	0	0	0.0	0.0	3.1	0.0	0.0	0.0	0.0	0.0	k:Bacteria p:Chloroflexi c:TK10 o:B07_WMSP1 f:FFCH4570
OTU.76uparse	1	0	1	0	0	0	0	0	0.1	0.0	1.5	0.0	0.0	0.0	0.0	0.0	k:Bacteria p:Planctomycetes c:Planctomycetia o:Planctomycetales f:Planctomycetaceae g:Planctomyces
OTU.2401uparse	0	0	0	2	0	0	0	0	0.0	0.0	0.0	0.6	0.0	0.0	0.0	0.0	k:Bacteria p:Acidobacteria c:DA052 o:Ellin6513
OTU.1147uparse	0	0	0	2	0	0	0	0	0.0	0.0	0.0	0.6	0.0	0.0	0.0	0.0	k:Bacteria p:Actinobacteria c:Acidimicrobiia o:Acidimicrobiales
OTU.28uparse	1	0	1	0	0	0	0	0	0.1	0.0	1.5	0.0	0.0	0.0	0.0	0.0	k:Bacteria p:Actinobacteria c:Actinobacteria o:Bifidobacteriales f:Bifidobacteriaceae g:Bifidobacterium s:
OTU.15394uparse	0	2	0	0	0	0	0	0	0.0	0.5	0.0	0.0	0.0	0.0	0.0	0.0	k:Bacteria p:Bacteroidetes c:Bacteroidia o:Bacteroidales f:S24-7
OTU.3260uparse	2	0	0	0	0	0	0	0	0.2	0.0	0.0	0.0	0.0	0.0	0.0	0.0	k:Bacteria p:Firmicutes c:Bacilli o:Lactobacillales f:Lactobacillaceae g:Lactobacillus s:
OTU.2451uparse	2	0	0	0	0	0	0	0	0.2	0.0	0.0	0.0	0.0	0.0	0.0	0.0	k:Bacteria p:Firmicutes c:Clostridia o:Clostridiales f:Ruminococcaceae g:Oscillospira s:
OTU.415uparse	0	0	0	2	0	0	0	0	0.0	0.0	0.0	0.6	0.0	0.0	0.0	0.0	k:Bacteria p:Proteobacteria c:Gammaproteobacteria o:Pseudomonadales f:Moraxellaceae g:Acinetobacter s:
OTU.15uparse	0	1	0	0	0	0	0	0	0.0	0.2	0.0	0.0	0.0	0.0	0.0	0.0	k:Bacteria p:Proteobacteria c:Betaproteobacteria o:Burkholderiales f:Comamonadaceae
OTU.3186uparse	1	0	0	0	0	0	0	0	0.1	0.0	0.0	0.0	0.0	0.0	0.0	0.0	k:Bacteria p:Proteobacteria c:Alphaproteobacteria o:Rhizobiales f:
OTU.88uparse	0	0	1	0	0	0	0	0	0.0	0.0	1.5	0.0	0.0	0.0	0.0	0.0	k:Bacteria p:Actinobacteria c:Actinobacteria o:Actinomycetales f:Mycobacteriaceae g:Mycobacterium s:
OTU.32uparse	0	0	1	0	0	0	0	0	0.0	0.0	1.5	0.0	0.0	0.0	0.0	0.0	k:Bacteria p:Actinobacteria c:Actinobacteria o:Actinomycetales f:
OTU.369uparse	0	0	0	1	0	0	0	0	0.0	0.0	0.0	0.3	0.0	0.0	0.0	0.0	k:Bacteria p:Planctomycetes c:Planctomycetia o:Gemmatales f:Gemmataceae
OTU.66uparse	0	0	1	0	0	0	0	0	0.0	0.0	1.5	0.0	0.0	0.0	0.0	0.0	k:Bacteria p:Acidobacteria c:Solibacteres o:Solibacterales f:Solibacteraceae g:CandidatusSolibacter s:
OTU.68uparse	0	0	0	0	1	0	0	0	0.0	0.0	0.0	0.0	3.2	0.0	0.0	0.0	k:Bacteria p:Proteobacteria c:Alphaproteobacteria o:Rhodospirillales f:Rhodospirillaceae
OTU.71uparse	0	0	1	0	0	0	0	0	0.0	0.0	1.5	0.0	0.0	0.0	0.0	0.0	k:Bacteria p:Actinobacteria c:Actinobacteria o:Actinomycetales f:Streptomycetaceae g:Streptomyces s:
OTU.3864uparse	0	0	1	0	0	0	0	0	0.0	0.0	1.5	0.0	0.0	0.0	0.0	0.0	k:Bacteria p:Proteobacteria c:Gammaproteobacteria o:Thiotrichales f:Piscirickettsiaceae
OTU.85uparse	1	0	0	0	0	0	0	0	0.1	0.0	0.0	0.0	0.0	0.0	0.0	0.0	k:Bacteria p:AD3 c:ABS-6 o: f:
OTU.4397uparse	1	0	0	0	0	0	0	0	0.1	0.0	0.0	0.0	0.0	0.0	0.0	0.0	k:Bacteria p:Acidobacteria c:Acidobacteriia o:Acidobacteriales f:Koribacteraceae
OTU.212uparse	0	0	1	0	0	0	0	0	0.0	0.0	1.5	0.0	0.0	0.0	0.0	0.0	k:Bacteria p:Verrucomicrobia c:[Pedosphaerae] o:[Pedosphaerales] f:[Pedosphaeraceae] g:Pedosphaera s:
OTU.10523uparse	0	0	1	0	0	0	0	0	0.0	0.0	1.5	0.0	0.0	0.0	0.0	0.0	k:Bacteria p:Proteobacteria c:Alphaproteobacteria o:Rhizobiales f:Hyphomicrobiaceae g:Rhodoplanes s:
OTU.2367uparse	1	0	0	0	0	0	0	0	0.1	0.0	0.0	0.0	0.0	0.0	0.0	0.0	k:Bacteria p:Proteobacteria c:Alphaproteobacteria o:Rhodospirillales f:Rhodospirillaceae
OTU.199uparse	0	0	0	1	0	0	0	0	0.0	0.0	0.0	0.3	0.0	0.0	0.0	0.0	k:Bacteria p:Proteobacteria c:Gammaproteobacteria o:Xanthomonadales f:Xanthomonadaceae
OTU.14519uparse	0	0	1	0	0	0	0	0	0.0	0.0	1.5	0.0	0.0	0.0	0.0	0.0	k:Bacteria p:Proteobacteria c:Alphaproteobacteria o:Rhodospirillales f:Rhodospirillaceae
OTU.57uparse	0	0	1	0	0	0	0	0	0.0	0.0	1.5	0.0	0.0	0.0	0.0	0.0	k:Bacteria p:Acidobacteria c:Acidobacteria-6 o:iii1-15 f:
OTU.438uparse	0	0	0	1	0	0	0	0	0.0	0.0	0.0	0.3	0.0	0.0	0.0	0.0	k:Bacteria p:Chloroflexi c:Anaerolineae o:SBR1031 f:oc28
OTU.591uparse	0	0	1	0	0	0	0	0	0.0	0.0	1.5	0.0	0.0	0.0	0.0	0.0	k:Bacteria p:OD1 c:SM2F11 o: f:
OTU.62uparse	0	0	1	0	0	0	0	0	0.0	0.0	1.5	0.0	0.0	0.0	0.0	0.0	k:Bacteria p:Acidobacteria c:DA052 o:Ellin6513 f:

OTU.111uparse	0	0	1	0	0	0	0	0	0.0	0.0	1.5	0.0	0.0	0.0	0.0	0.0	k:Bacteria p:Proteobacteria c:Alphaproteobacteria o:Ellin329 f:
OTU.246uparse	0	0	1	0	0	0	0	0	0.0	0.0	1.5	0.0	0.0	0.0	0.0	0.0	k:Bacteria p:Verrucomicrobia c:[Spartobacteria] o:[Chthoniobacterales] f:[Chthoniobacteraceae] g:CandidatusXiphinematobacter s:
OTU.580uparse	0	0	1	0	0	0	0	0	0.0	0.0	1.5	0.0	0.0	0.0	0.0	0.0	k:Bacteria p:Acidobacteria c:Acidobacteriia o:Acidobacteriales f:Koribacteraceae g:CandidatusKoribacter s:
OTU.5099uparse	0	0	1	0	0	0	0	0	0.0	0.0	1.5	0.0	0.0	0.0	0.0	0.0	k:Bacteria p:Proteobacteria c:Alphaproteobacteria o:Rhizobiales f:
OTU.177uparse	0	1	0	0	0	0	0	0	0.0	0.2	0.0	0.0	0.0	0.0	0.0	0.0	k:Bacteria p:Bacteroidetes c:Sphingobacteriia o:Sphingobacteriales f:
OTU.4327uparse	0	1	0	0	0	0	0	0	0.0	0.2	0.0	0.0	0.0	0.0	0.0	0.0	k:Bacteria p:Actinobacteria c:Acidimicrobiia o:Acidimicrobiales f:
OTU.1729uparse	0	0	0	0	0	1	0	0	0.0	0.0	0.0	0.0	0.0	0.6	0.0	0.0	k:Bacteria p:Chloroflexi c:Ktedonobacteria o:JG30-KF-AS9 f:
OTU.133uparse	0	1	0	0	0	0	0	0	0.0	0.2	0.0	0.0	0.0	0.0	0.0	0.0	k:Bacteria p:AD3 c:ABS-6 o: f:
OTU.149uparse	0	0	1	0	0	0	0	0	0.0	0.0	1.5	0.0	0.0	0.0	0.0	0.0	k:Bacteria p:Gemmatimonadetes c:Gemm-1 o: f:
OTU.110uparse	0	0	1	0	0	0	0	0	0.0	0.0	1.5	0.0	0.0	0.0	0.0	0.0	k:Bacteria p:Firmicutes c:Clostridia o:Clostridiales f:Clostridiaceae g:SMB53 s:
OTU.914uparse	0	0	0	0	0	1	0	0	0.0	0.0	0.0	0.0	0.0	0.6	0.0	0.0	k:Bacteria p:Proteobacteria c:Betaproteobacteria o:Burkholderiales f:Burkholderiaceae g:Burkholderia s:
OTU.1241uparse	0	1	0	0	0	0	0	0	0.0	0.2	0.0	0.0	0.0	0.0	0.0	0.0	k:Bacteria p:Verrucomicrobia c:[Pedosphaerae] o:[Pedosphaerales] f:[Pedosphaeraceae]
OTU.977uparse	0	0	0	1	0	0	0	0	0.0	0.0	0.0	0.3	0.0	0.0	0.0	0.0	k:Bacteria p:Proteobacteria c:Betaproteobacteria o: f:
OTU.1003uparse	0	0	1	0	0	0	0	0	0.0	0.0	1.5	0.0	0.0	0.0	0.0	0.0	k:Bacteria p:Proteobacteria c:Deltaproteobacteria o:Myxococcales f:Haliangiaceae
OTU.963uparse	0	0	1	0	0	0	0	0	0.0	0.0	1.5	0.0	0.0	0.0	0.0	0.0	k:Bacteria p:Acidobacteria c:Solibacteres o:JH-WHS99 f:
OTU.3832uparse	0	0	0	1	0	0	0	0	0.0	0.0	0.0	0.3	0.0	0.0	0.0	0.0	k:Bacteria p:Chloroflexi c:Ktedonobacteria o:Elev-1554 f:
OTU.3818uparse	0	0	0	0	0	1	0	0	0.0	0.0	0.0	0.0	0.0	0.6	0.0	0.0	k:Bacteria p:Acidobacteria c:Acidobacteria-6 o:CCU21 f:
OTU.4795uparse	0	0	1	0	0	0	0	0	0.0	0.0	1.5	0.0	0.0	0.0	0.0	0.0	k:Bacteria p:Verrucomicrobia c:[Pedosphaerae] o:[Pedosphaerales] f:
OTU.13909uparse	0	1	0	0	0	0	0	0	0.0	0.2	0.0	0.0	0.0	0.0	0.0	0.0	k:Bacteria p:Firmicutes c:Bacilli o:Bacillales f:Staphylococcaceae g:Staphylococcus s:
OTU.14695uparse	1	0	0	0	0	0	0	0	0.1	0.0	0.0	0.0	0.0	0.0	0.0	0.0	k:Bacteria p:Actinobacteria c:Actinobacteria o:Actinomycetales f:Dermacoccaceae g:Dermacoccus s:
OTU.282uparse	0	0	1	0	0	0	0	0	0.0	0.0	1.5	0.0	0.0	0.0	0.0	0.0	k:Bacteria p:Bacteroidetes c:Flavobacteriia o:Flavobacteriales f:Flavobacteriaceae g:Flavobacterium s:
OTU.10034uparse	0	1	0	0	0	0	0	0	0.0	0.2	0.0	0.0	0.0	0.0	0.0	0.0	k:Bacteria p:Firmicutes c:Bacilli o:Lactobacillales f:Aerococcaceae
OTU.40uparse	0	0	1	0	0	0	0	0	0.0	0.0	1.5	0.0	0.0	0.0	0.0	0.0	k:Bacteria p:Proteobacteria c:Gammaproteobacteria o:Pseudomonadales f:Moraxellaceae g:Acinetobacter s:
OTU.4550uparse	0	0	0	0	0	0	1	0	0.0	0.0	0.0	0.0	0.0	0.0	50.0	0.0	k:Bacteria p:Bacteroidetes c:Bacteroidia o:Bacteroidales f:Prevotellaceae g:Prevotella s:stercorea
